# A Systematic Approach to Exergy Efficiency of Steady-Flow Systems

**DOI:** 10.3390/e27111108

**Published:** 2025-10-26

**Authors:** Yunus A. Çengel, Mehmet Kanoğlu

**Affiliations:** 1 Department of Mechanical Engineering, University of Nevada, Reno, NV 89557, USA; 2 Department of Mechanical Engineering, Alanya Alaaddin Keykubat University, Antalya 07425, Türkiye; mehmet.kanoglu@alanya.edu.tr

**Keywords:** thermodynamics, exergy, exergy efficiency, second-law efficiency, exergy analysis, steady-flow devices, heat engines, refrigerators

## Abstract

Exergy efficiency is a measure of thermodynamic perfection. A device that operates reversibly has an exergy efficiency of 100 percent and is said to be thermodynamically perfect. A reversible process involves zero entropy generation and thus zero exergy destruction since *X*_destroyed_ = *T*_0_*S*_gen_. Exergy efficiency is generally defined as the ratio of exergy output to exergy input *η*_ex_ = *X*_output_/*X*_input_ = 1 − (*X*_destroyed_ + *X*_loss_)/*X*_input_ or the ratio of exergy recovered to exergy expended *η*_ex_ = *X*_recovered_/*X*_expended_ = 1 − *X*_destroyed_/*X*_expended_. In this paper, exergy efficiency relations are obtained first for a general steady-flow system using both approaches. Then, explicit general relations are obtained for common steady-flow devices, such as turbines, compressors, pumps, nozzles, diffusers, valves and heat exchangers, as well as heat engines, refrigerators, and heat pumps. For power and refrigeration cycles, five different forms of exergy efficiency relations are developed, and their equivalence is demonstrated. With the unified approach presented here and the insights provided, the controversy and confusion associated with different exergy efficiency definitions are largely alleviated.

## 1. Introduction

Exergy is the work potential of energy. It is the maximum work that a system can produce in a given environment when undergoing a totally reversible process from its initial state to the state of the environment [1,2,3,4,5,6,7,8,9,10]. In exergy calculations, it is understood that any heat, work, and mass interactions can occur only between the system and its environment. The word *exergy* was coined by the Swedish engineer Zoran Rant in 1956 as a concise universal term to replace the ambiguous phrases *availability*, *available energy*, *usable energy*, and *available work* in use at the time by combining the Greek word *ergon* (meaning *work*) with the prefix *ex-* (meaning *out of*) and the suffix *-y* (used to form nouns, as in *energy*), and was described as *technical work capacity* [11]. As such, exergy literally means *capacity to do work* or *work potential of energy*.

A key parameter of second-law analysis of energy systems is *exergy efficiency*, also known as second-law efficiency [1], exergetic efficiency [4], rational efficiency [6], and effectiveness [9]. We prefer the term *exergy efficiency* to *exergetic efficiency* because it is simpler and parallels the commonly used phrase *energy efficiency*. Exergy efficiency is a measure of an energy system’s actual performance relative to the thermodynamically best possible performance, which occurs under reversible conditions characterized by zero entropy generation or zero exergy destruction. Therefore, the exergy efficiency of all reversible processes or devices is 100 percent. A system with *low exergy destruction* and thus *high exergy efficiency* approximates reversible operation more closely.

Different forms of exergy efficiency have been defined and discussed by several researchers. Kotas [6], Szargut et al. [7], and Moran [10] provided extensive coverage of exergy concept and defined exergy efficiencies for various processes. Szargut [7] provided an exergy efficiency relation as the ratio of useful exergy effect to the consumption of driving exergy, noting that the use of this relation required proper identification of the terms in the numerator and the denominator. Sciubba and Wall [12] provided a summary of the various exergy efficiencies. Cornelissen [13] presented three different definitions of exergy efficiency for steady-flow processes. The first is the total output exergy flow over total input exergy. The second is based on Kotas [6], which is the desired exergy output over the exergy used. The third one is based on Brodyansky et al. [14], that is the total outgoing exergy flow minus the untransformed components of exergy to the total incoming exergy flow minus the untransformed components of exergy. Lior and Zhang [15] attempted to clarify different performance criteria for energy systems considering simultaneous energy interactions of work, heating, and cooling. They made distinction between exergy efficiency and second-law efficiency and discussed multiple definitions considering different devices. Marmolejo-Correa and Gundersen [16] focused on low-temperature processes including natural gas liquefaction to discuss various definitions of exergy efficiency. Nguyen et al. [17] also discussed various ways of defining exergy efficiency and developed a component-by-component exergy efficiency relation for offshore oil and gas processing.

There are many studies in the literature on energy systems that make use of exergy efficiency as part of the analysis. For example, Paul et al. [18] examined the exergy characteristics of a CI engine and calculated exergy efficiency using the relation *η*_ex_ = (Shaft power)/(Inlet air exergy + Chemical exergy of fuel). Chen et al. [19] evaluated a modified ejector-enhanced refrigeration cycle using energy and exergy analysis and determined exergy efficiency from *η*_ex_ = 1 − (Total exergy destroyed)/(Compressor power). Gurbuz et al. [20] calculated exergy efficiency of a diffusion–absorption refrigeration system using a similar relation, *η*_ex_ = 1 − (Total exergy destroyed)/(Exergy input), but it was unclear what exactly the exergy input represented in the system. Molina-Salas et al. [21] uses two different relations for exergy efficiency of a simple offshore oscillating water column device—*η*_ex_ = (Exergy output)/(Exergy input) and *η*_ex_ = 1 − (Exergy destruction at products)/(Exergy destruction at sources). These two relations do not seem to be consistent with each other. In their exergy analysis of a modern turboprop engine, Kirmizi et al. [22] acknowledged several ways for expressing exergy efficiency and formulated it as *η*_ex_ = (Product exergy)/(Fuel exergy) = 1 − (Exergy destroyed)/(Fuel exergy). Kanoglu [23] used two different exergy efficiency relations when evaluating the performance of a geothermal power plant. Kanoglu [24] developed exergy efficiency relations for a multistage cascade refrigeration cycle used for natural gas liquefaction.

The performance of various energy systems is analyzed and optimized using second-law analysis, which includes exergy efficiency. Examples from the literature include solar-driven water gasification systems [25], hybrid solar–geothermal polygeneration systems [26], geothermal cogeneration systems [27], combined cycle power plants [28], regenerative closed Brayton cycles [29], reversible absorption heat pumps [30], and waste heat recovery [31].

Brötz et al. [32] defined exergy efficiency of a fan as the ratio of the exergy change in the fluid across the fan to the shaft power input and compared it with the isentropic efficiency. Zulkefal et al. [33] performed an analysis of a methanol production plant using the exergy method and calculated exergy efficiencies of all components including mixing chambers, heat exchangers, reactors, and valves using a single exergy output to exergy input formula. Hecht et al. [34] reviewed ecologically regenerative building systems through exergy efficiency without introducing any specific definition for exergy efficiency. In their analysis of industrial pneumatic systems, Zhao et al. [35] used the fuel exergy–product exergy approach in defining exergy efficiencies of various components such as an air compressor, an aftercooler, and a pressure-reducing valve. Exergy efficiency is also defined and calculated for other energy conversion systems including integrated natural gas liquids and liquefied natural gas processes [36], drying plants [37], steam power plants [38], and ground-source heat pumps [39]. The evaluation of exergy efficiency as part of a performance assessment of energy systems has been the topic of numerous other articles [40,41,42,43,44,45,46,47,48,49,50,51,52,53,54].

Exergy or second-law analysis is often considered a theoretical topic rather than a practical tool. One reason for this perception is the differing viewpoints on how exergy efficiency should be defined. These differing definitions result in inconsistencies and confusion. Consequently, there are different definitions of exergy efficiency in the literature, and thus the exergy efficiency of a process can differ depending on which definition is used. The challenge here is to choose the most suitable definition for the situation at hand. General definitions and explicit relations of exergy efficiency for closed and unsteady-flow systems were developed in a prior study [55]. In this study, a general definition of exergy efficiency is developed for steady-flow systems. Then, specific exergy definitions are obtained for common steady-flow devices, such as turbines, compressors, pumps, nozzles, diffusers, valves, and heat exchangers, as well as heat engines, refrigerators, and heat pumps, by simplifying the general definition for the situation at hand.

## 2. Definitions of Exergy Efficiency

Exergy efficiency is intended to serve as a measure of approximation to reversible operation; thus, its value should range from zero in the worst case (complete destruction of exergy) to one in the best case (no destruction of exergy). Even at the fundamental level, there is no consensus on how exergy efficiency should be defined. Exergy efficiency, *η*_ex_, is commonly expressed as the ratio of the exergy output *X*_out_ to the exergy input *X*_in_ [56],

(1) $$\eta_{\mathrm{ex}}=\frac{\mathrm{Exergy}\;\mathrm{output}}{\mathrm{Exergy}\;\mathrm{input}}=\frac{X_{\mathrm{out}}}{X_{\mathrm{in}}}=1-\frac{X_{\mathrm{destroyed}}+X_{\mathrm{loss}}}{X_{\mathrm{in}}}$$

This definition compares the exergy that leaves the system as the *desirable output* or *valuable product* to the exergy supplied to the system as the *required input* or *invested commodity* in the currency of exergy. The exergy inputs and outputs may involve exergy transfer by heat transfer, work, and mass flow. But usually, the exergy change in a fluid stream as it passes through the control volume is taken as the exergy input or output associated with that fluid stream rather than the exergy at the inlet or exit. In a steam turbine, for example, the invested exergy is the decrease in the exergy of the steam between the inlet and exit of the turbine rather than the exergy of steam at the turbine inlet. Likewise, in an air compressor, the valuable product associated with the air stream is the increase in the exergy of the air between the exit and the inlet of the compressor rather than the exergy of air at the compressor exit. This way, the exergy efficiency of a turbine represents the fraction of the steam exergy consumed in the turbine that is converted to work while the exergy efficiency of a compressor represents the fraction of power consumed that is stored in the air as exergy. Also, the sum of exergy destroyed and exergy loss is equal to the difference between exergy input and output, *X*_destroyed_ + *X*_loss_ = *X*_in_ − *X*_out_. The term *X*_loss_ is not included in the second part of the exergy efficiency expression in some formulations [57].

There is often ambiguity about what should be considered *exergy output* versus *exergy loss*, and how the two are distinguished. The distinction depends on the system’s purpose and role in the overall process. Exergy carried away as heat lost to the surroundings is clearly an exergy loss, while exergy transferred to a preheater or cogeneration unit is not. Similarly, the kinetic, chemical, and thermal exergies of gases released to the environment represent exergy loss. Consistent exergy accounting is necessary to avoid double-counting.

Both *exergy destruction* and *exergy loss* signify wasted exergy, unless the lost part is utilized as input for another system. Whether wasted exergy is classified as loss or destruction depends on how the system boundaries are defined. For example, exergy carried away with heat loss from a device is considered *exergy loss* if the device itself is defined as the system. However, if the *extended system* that includes the system and its immediate surroundings is taken as the system, that same exergy is regarded as *exergy destruction*, since the exergy loss associated with heat loss will be destroyed in the immediate surroundings. Similarly, the thermal, chemical, and kinetic exergy contained in exhaust gases is treated as *exergy loss* when the physical device is taken as the system. In contrast, it is taken as *exergy destruction* when the *extended system* is taken as the system, because the exhaust gases eventually reach environmental conditions of temperature, pressure, velocity, and composition at the boundary. Since exergy loss is ultimately destroyed, it is often more practical to work with the extended system and treat both quantities as exergy destruction, simplifying the analysis.

An alternative form of Equation (1) is expressed as follows [4]:

(2) $$\eta_{\mathrm{ex}}=\frac{\mathrm{Product}}{\mathrm{Fuel}}=\frac{X_{\mathrm{product}}}{X_{\mathrm{fuel}}}=1-\frac{X_{\mathrm{destroyed}}+X_{\mathrm{loss}}}{X_{\mathrm{fuel}}}$$

where *product* represents the *desired output* of the system in terms of *exergy produced*, reflecting the purpose of purchasing the system while the *fuel* represents the *required exergy resource input* and it is not limited to an actual fuel. Equations (1) and (2), in general, are not identical since “fuel” and “product” must be defined in a rational way for each individual device or process and they may not be equal to the definitions of the “exergy input” and “exergy output”. The two approaches become identical when the *fuel exergy* is interpreted as the exergy input and the *product exergy* is taken as the exergy output. Of the two definitions above, we will refer to the first one in the analysis since the terms *exergy input* and *exergy output* are more intuitive and general than the terms *fuel* and *product*.

Another form of Equation (1) for exergy efficiency is given for steady-flow systems as the ratio of the desired exergy output to the necessary exergy input as follows [6]:

(3) $$\eta_{\mathrm{ex}}=\frac{\mathrm{Desired}\;\mathrm{exergy}\;\mathrm{output}}{\mathrm{Necessary}\;\mathrm{exergy}\;\mathrm{input}}=\frac{X_{\mathrm{out}}}{X_{\mathrm{in}}}=1-\frac{X_{\mathrm{destroyed}}}{X_{\mathrm{in}}}$$

Exergy inputs and outputs may involve exergy transfer by heat transfer, work, and mass flow. But as stated above, the *exergy change in a* fluid stream as it passes through the control volume is usually taken as the exergy input or output associated with that fluid stream. The purpose of the system being analyzed is the determining factor when an exergy transfer is identified as input or output. In this formulation, authors use name *rational efficiency ψ* for exergy efficiency and *irreversibility rate* $\dot{I}$ for exergy destruction.

Exergy efficiency is also expressed in a general form for steady-flow systems as well as closed and unsteady-flow systems as the ratio of the *exergy expended* to *exergy recovered* as follows [1]:

(4) $$\eta_{\mathrm{ex}}=\frac{\mathrm{Exergy}\;\mathrm{recovered}}{\mathrm{Exergy}\;\mathrm{expended}}=\frac{X_{\mathrm{recovered}}}{X_{\mathrm{expended}}}=1-\frac{X_{\mathrm{destroyed}}}{X_{\mathrm{expended}}}$$

where $X_{\mathrm{destroyed}}=X_{\mathrm{expended}}-X_{\mathrm{recovered}}$ is the difference between exergy expended and recovered. This expression differs from Equations (1) and (2) since it does not involve the term *exergy loss*. When the physical device is taken as the system, *recovered exergy* includes the exergy that is eventually lost (associated with heat loss and mass discharge). Therefore, deviation in exergy efficiency from 100% serves as a measure of *exergy destruction* within the system boundaries.

Decisions on what is to be included as expended and recovered exergy or exergy input and output can be made by considering the system’s intended purpose and the specifics of the process. Therefore, it is important to decide early in the analysis what constitutes exergy input–output or exergy expended–recovered by considering the purpose of the system and what is required as inputs. Different assessments result in different exergy efficiency relations, so the objective should be to come up with the most meaningful definition that provides the best insight.

For steady-flow devices that are intended to produce work such as turbines or to pressurize a fluid by consuming work such as compressors, pumps, and fans, exergy efficiency is commonly defined in reference to *reversible work W*_rev_ as follows:

(5) $$\eta_{\mathrm{ex},W\_\mathrm{out}}=\frac{W_{\mathrm{act},\mathrm{out}}}{W_{\mathrm{rev},\mathrm{out}}}\;\;\mathrm{and}\;\;\eta_{\mathrm{ex},W\_\mathrm{in}}=\frac{W_{\mathrm{rev},\mathrm{in}}}{W_{\mathrm{act},\mathrm{in}}}$$

where *W*_act_ is the actual work.

To accurately identify exergy interactions between the system and its surroundings, the system in question must be well-defined. For a heat engine, the *expended exergy* is the exergy of the heat transferred to the engine, and the *recovered exergy* is the net work produced by the engine and the exergy of heat rejected. If heat is rejected at the environmental temperature, the rejected heat’s exergy is zero. In this case, exergy expended is equal to exergy supplied by heat, and exergy recovered is equal to net work output. For a refrigerator or heat pump, the *exergy expended* is usually the work consumed, which is identical to the exergy input. *Recovered exergy* is the exergy of the heat transferred to the high-temperature medium for a heat pump and the exergy of the heat transferred from the low-temperature medium for a refrigerator.

## 3. Novelty and Methodology

This paper represents an original contribution to the literature on the definition of exergy efficiency of systems or devices that operate steadily and clarifies some common misconceptions. Exergy efficiency relations for many steady-flow devices are widely available in thermodynamics textbooks and are commonly used in papers on second-law analysis. However, inconsistencies and inaccuracies are prevalent. For example, exergy efficiency of heat exchangers is defined differently by different authors, and some of these definitions become inappropriate when temperatures below the environment temperature are involved. Similarly, the exergy efficiency relation for adiabatic turbines is properly defined, but this is not always the case for turbines with a heat loss. Additionally, there is not a consistent approach for the evaluation of the exergy efficiency of valves, diffusers, and nozzles.

This comprehensive paper systematically and consistently re-develops the exergy efficiencies of all common steady-flow devices from the fundamentals, bringing uniformity to the treatment of exergy efficiency of all steady-flow devices. This is achieved by first developing a general steady-flow exergy efficiency relation and then simplifying it for specific devices. This provides a common base for all devices that are traceable to the fundamentals. Any limitations on the specific exergy efficiency relations are indicated. The exergy relations for power and refrigeration cycles are also redeveloped from the fundamentals, and it is demonstrated that five seemingly unrelated exergy efficiency relations used for power cycles and refrigeration cycles are equivalent to each other. This provides valuable new insight into the exergy analysis of power plants, refrigerators, and heat pumps. The paper also provides the necessary tools for determining the most appropriate exergy efficiency relation for the situation at hand among all alternatives.

The root of the problems associated with exergy efficiency relations appears to be taking the relation ${\eta_{\mathrm{ex}}=X}_{\mathrm{out}}/X_{\mathrm{in}}$ as the sole general definition of exergy efficiency and applying it to all systems. While this fundamental relation works well for most steady-flow systems, it does not work for all of them. The alternative general definition ${\eta_{\mathrm{ex}}=X}_{\mathrm{recovered}}/X_{\mathrm{expended}}$ works well for closed and unsteady-flow systems as well as steady-flow systems. The corresponding equivalent relations ${\eta_{\mathrm{ex}}=1-(X}_{\mathrm{destroyed}}+X_{\mathrm{loss}})/X_{\mathrm{in}}$ and ${\eta_{\mathrm{ex}}=1-X}_{\mathrm{destroyed}}/X_{\mathrm{expended}}$ also work well. There is often ambiguity as to what constitutes exergy input, exergy output, exergy expended, and exergy recovered. These terms should be properly identified by considering the particulars of the analyzed system and keeping in mind its intended task.

**Figure 1:**
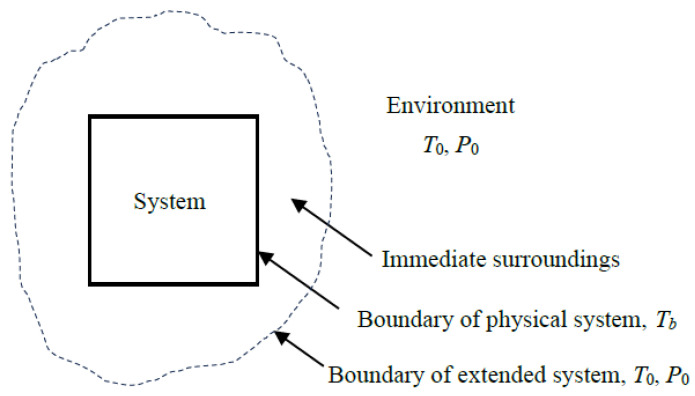
Extended system that includes the system and its immediate surroundings.

Most differences in defining exergy efficiency stem from how the exergy transfer associated with heat loss and expelled mass is treated—whether it is categorized as *exergy loss* or *exergy destruction*. This ambiguity can be resolved by applying the concept of the *extended system*, consisting of the system and its immediate surroundings where temperature and concentration gradients exist (Figure 1). The extended system includes the temperature and concentration gradient zones, and the boundary temperature, pressure, and concentration values are those of the environment.

For example, when a turbine with a heat loss *Q*_loss_ to the surroundings is taken as the system, the outer surface of the turbine constitutes the system boundary, and the exergy transfer associated with heat loss (*X_Q_*__loss_) is determined from *X_Q_*__loss_ = (1 − *T*_0_/*T_b_*)*Q*_loss_ where *T_b_* is the average boundary temperature. The exergy destroyed within the immediate surroundings can be determined from $X_{\mathrm{destroyed}}=T_{0}S_{\mathrm{gen}}$ where *S*_gen_ is entropy generation calculated from the entropy balance on the immediate surroundings, $S_{\mathrm{gen}}=Q_{loss}/T_{0}-Q_{loss}/T_{b}$.

The term *X_Q_*__loss_ is part of the *exergy output* for the turbine when the turbine is taken as the system. Unlike other exergy output terms, such as the shaft work, it is properly called *exergy loss* since the exergy associated with heat loss is unlikely to be recovered and utilized for a useful purpose elsewhere. As a result, exergy loss is usually destined to be exergy destroyed, but the destruction takes place outside the system boundaries. To properly account for the exergy destruction that occurs in the immediate surroundings, it should be clear whether the immediate surroundings of the system are included as part of the system or as part of the surroundings. The *extended system* approach can also be used for systems whose outer surfaces are covered with thermoelectric generators by simply including the work produced by them as part of the exergy output.

The treatment of *X_Q_*__loss_ is a source of confusion since some consider it part of exergy output and others consider it part of exergy destruction when evaluating exergy efficiency. The extended system analysis bypasses this confusion by doing away with the concept of *exergy loss* and treating the potential destruction of exergy as an actuality. In the case of the *extended system*, the exergy transfer associated with heat loss and purged mass is zero since the exergy content of thermal energy and mass at the environmental conditions is zero. The extended-system approach simplifies the analysis and eliminates the need to distinguish between exergy loss and exergy destruction by including exergy loss associated with heat loss and purged substances in exergy destruction. The exergy efficiency evaluated this way is also more realistic since it properly accounts for all exergy destruction associated with the process, including the exergy destroyed within the immediate surroundings, which is unavoidable. Therefore, the exergy efficiency of the *extended system* is equivalent to the exergy efficiency of the *process*. For *adiabatic* systems, the choice between an actual and an extended system is irrelevant because both yield the same result when the immediate surroundings shrink to zero.

When a system discharges substances into the environment as part of its mass outflow, along with heat losses, the immediate surroundings form a region with concentration and temperature gradients between the system and the environment. The purged matter mixes with the surroundings such that the composition at the boundary of the extended system becomes identical to that of the environment. In other words, each component of the discharged mass transitions from its initial state to the state of the environment (the dead state), including changes in concentration. As a result, the exergy of the purged mass, including its chemical exergy, becomes zero at the extended system boundary, *X*_purged mass_ = 0. When the physical system is taken as the system, the exergy transfer associated with purged mass constitutes an exergy loss, *X*_loss, purged mass_. The quantity of *X*_purged mass_ or *X*_loss, purged mass_ represents the work potential of the purged mass at the boundary of the physical system which is eventually destroyed in the immediate surroundings.

In the analysis below, we develop exergy efficiency relations for the *physical system* by considering exergy transfer by heat loss *X_Q_*__loss_ as part of the exergy recovered in the exergy expended–recovered approach. In the exergy input–output approach, *X_Q_*__loss_ is grouped with destroyed exergy and not with exergy output. The exergy efficiency relations for the corresponding *extended system* or process can be obtained by setting this term equal to zero, *X_Q_*__loss_ = 0 in both approaches. Exergy transfer associated with mass flow purged into the environment, if any, does not appear explicitly in the formulations below since it is treated as one of the fluid streams exiting the system. The exergy associated with purged mass is zero for an extended system.

## 4. Exergy Efficiency for a General Steady-Flow System

Steady-flow systems are control volumes characterized by *no change with time* at any location within the control volume boundaries. As a result, the change in the energy, entropy, and exergy contents of a system during steady operation is zero, Δ*E*_sys_ = 0, Δ*S*_sys_ = 0, and Δ*X*_sys_ = 0. Many engineering systems such as power plants, refrigerators, heaters, turbines, compressors, pumps, heat exchangers, mixing chambers, nozzles, diffusers, and valves are designed for steady operation and are usually analyzed on that basis. In this section, we develop exergy efficiency relations for a general steady-flow system using the approaches given above.

**Figure 2:**
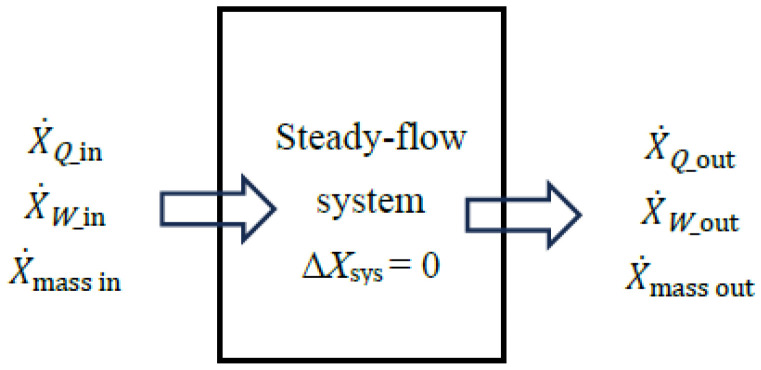
Exergy interactions associated with a steady-flow system.

A steady-flow system may involve exergy transfer into or out of the system by heat, work, and mass flow (Figure 2). However, unlike a closed or unsteady-flow system, it cannot involve a change in its exergy content, and thus the term Δ*X*_sys_ does not appear in exergy–efficiency relations. The sum of exergy transfer into a steady-flow system by heat, work, and mass constitutes *exergy expended* while the sum of the exergy transfer out of the system by heat, work, and mass constitutes *exergy recovered*. The difference between the two is *exergy destroyed*.

In the analysis, the exergy resources utilized, such as heat transfer, mechanical or electrical work supply, or incoming fluid streams, are first identified. Work consumed by the system is part of exergy expended in its entirety. When the exergy source is a *fluid stream*, the exergy expended is the difference in exergy between the fluid’s inlet and exit states. When a fluid stream leaves the system at the dead state and thus with zero exit exergy, exergy expended associated with the stream is its inlet exergy. The same is true for heat input when all heat loss occurs at the environmental temperature.

*Energy* can exist in many forms such as thermal, mechanical, kinetic, potential, electric, magnetic, chemical, and nuclear, and their sum constitutes the *total energy*, *E* (or *e* on a unit mass basis) of a system. Each form of energy has *exergy* (work potential), and the sum of the exergies associated with each relevant form of energy gives the *total exergy X* (or *x* on a unit mass basis) of the system.

Kinetic and potential energies can be completely converted to mechanical work, and thus they are properly categorized as *mechanical energy*. Then, exergy *x* associated with kinetic and potential energies are as follows:

(6) $$x_{ke}=ke=V^{2}/2\;\;and\;x_{pe}=pe=gz\;$$

Here, *V* is the velocity of the system relative to the environment, *g* is the gravitational acceleration, and *z* is the elevation in the system relative to a reference level in the environment. For rotating bodies, kinetic energy includes *rotational* kinetic energy.

In the absence of electric, magnetic, and surface tension effects, the total energy of a flowing fluid consists of thermal energy represented by enthalpy *h* (*u* + *Pv* or internal energy + flow energy), kinetic energy ke, and potential energy pe as follows:

(7) $$e_{flow}=h+ke+pe=h+V^{2}/2+gz$$

The *exergy* associated with the energy of a flowing fluid, called *flow exergy x*_flow_, is obtained by undertaking a reversible process from the given state (no subscript) to the state of the environment (the dead state with “0” subscript) with the following result:

(8) $$x_{flow}=\left(h-h_{0}\right)-T_{0}\left(s-s_{0}\right)+ke+pe$$

where *h*_0_ and *s*_0_ are the enthalpy and entropy of the fluid stream *at the state of the environment*, respectively. Note that the thermal exergy of the flowing fluid is zero at the temperature and pressure of the environment, and thus *x*_flow_ expression represents the *useful work potential* (i.e., *exergy*) of the system at the given state per unit mass.

For a *fluid stream* of mass flow rate $\dot{m}$ entering a steady-flow system at state 1 and exiting at state 2, the exergy flow rate at the inlet and the exergy flow rate difference between the inlet and exit are expressed as follows [1]:

(9) $${\dot{X}}_{\mathrm{mass},1}={\dot{X}}_{\mathrm{flow}1}=\dot{m}x_{\mathrm{flow},1}=\dot{m}[\left(h_{1}-h_{0}\right)-T_{0}\left(s_{1}-s_{0}\right)+{\mathrm{ke}}_{1}+{\mathrm{pe}}_{1}]$$

$$∆{\dot{X}}_{\mathrm{flow}}={\dot{X}}_{\mathrm{mass},2}-{\dot{X}}_{\mathrm{mass},1}=\dot{m}\left(x_{\mathrm{flow},2}-x_{\mathrm{flow},1}\right)$$

or

(10) $$∆{\dot{X}}_{\mathrm{flow}}=\dot{m}[\left(h_{2}-h_{1}\right)-T_{0}\left(s_{2}-s_{1}\right)+\left({\mathrm{ke}}_{2}-{\mathrm{ke}}_{1}\right)+({\mathrm{pe}}_{2}-{\mathrm{pe}}_{1})]$$

Exergy transfer associated with *shaft* and *electrical work* is the work itself while exergy transfer associated with *heat transfer* at the system boundary at temperature *T_b_* is the amount of heat transfer *Q* multiplied by Carnot efficiency,

(11) $${\dot{X}}_{W}=\dot{W}\;(\mathrm{shaft}\;\mathrm{and}\;\mathrm{electrical}\;\mathrm{work})$$

(12) $${\dot{X}}_{Q}=\left(1-\frac{T_{0}}{T_{b}}\right)\dot{Q}$$

In Equation (12), the term 1 − *T*_0_/*T_b_* is the *Carnot efficiency* (also called *Carnot factor*) which is the thermal efficiency of a totally reversible Carnot heat engine that operates between a heat source at *T_s_* and the environment at *T*_0_. It represents the maximum work that can be produced for a given amount of heat input. Here, the source temperature *T_s_* is replaced by *T_b_* in order to express exergy transfer associated by heat transfer that takes place at the boundary temperature. The phrases *work* and *useful work* are synonymous for steady-flow systems since they do not involve moving boundary work.

The *direction* of exergy transfer with work is the same as the direction of work. The same is true for heat transfer for systems whose temperature is above the environmental temperature. However, the reverse is true for systems below the environmental temperature since heat transfer to such systems decreases their exergy. Therefore, it is not surprising that the equation above gives a *negative value* for exergy transfer ${\dot{X}}_{Q}$ associated with heat input $\dot{Q}$ when *T_b_* < *T*_0_. Restoring the cold system to its original state requires the reversing the heat gain process by removing heat in the amount of $\dot{Q}$ from the cold system. This can be performed by a Carnot refrigerator operating between the temperatures *T_b_* and *T*_0_. The required work input of this *reversible refrigerator* is as follows:

(13) $${\dot{W}}_{\mathrm{rev},\mathrm{in}}=\frac{{\dot{Q}}_{L}}{{COP}_{\mathrm{rev}}}=\frac{{\dot{Q}}_{L}}{T_{L}/(T_{H}-T_{L})}=\frac{(T_{H}-T_{L}){\dot{Q}}_{L}}{T_{L}}=\frac{(T_{0}-T_{b}){\dot{Q}}_{L}}{T_{b}}=-\left(1-\frac{T_{0}}{T_{b}}\right)Q_{L}=-{\dot{X}}_{Q\_L}$$

which is equivalent to the exergy transfer associated with heat transfer ${\dot{Q}}_{L}$ to the cold medium, except in the reverse direction. Exergy transfer with heat transfer becomes zero when *T_b_* = *T*_0_. Therefore, exergy transfer associated with heat loss from an *extended system* (system + its immediate surroundings where gradients occur) is zero since the *boundary temperature* of an extended system is equal to the environment temperature *T*_0_.

Considering that energy is *conserved*, entropy is *generated* (but never destroyed, *S*_gen_ ≥ 0), and exergy is *destroyed* (but never generated, *X*_destroyed_ ≥ 0), *energy, entropy, and exergy balances* for a *general system* undergoing any process can be written as follows:

(14) $$E_{in}-E_{out}=∆E_{sys}={\left(E_{2}-E_{1}\right)}_{sys}$$

(15) $$S_{in}-S_{out}+S_{gen}=∆S_{sys}={\left(S_{2}-S_{1}\right)}_{sys}$$

(16) $$X_{in}-X_{out}-X_{destroyed}=∆X_{sys}={\left(X_{2}-X_{1}\right)}_{sys}$$

*Entropy generation S*_gen_ and *exergy destruction X*_destroyed_ are related to each other by the following equation:

(17) $$X_{destroyed}=T_{0}S_{gen}$$

Both energy and exergy are transferred into and out of the system, in general, by heat, work, and mass flow while entropy is transferred by heat and mass (work is entropy-free). Therefore, *energy, entropy*, and *exergy balances* can be expressed more explicitly as follows:

(18) $$(Q_{in}-Q_{out})+(W_{in}-W_{out})={\left(E_{2}-E_{1}\right)}_{sys}$$

(19) $$(S_{Q,in}-S_{Q,out})+(S_{mass,in}-S_{mass,out})+S_{gen}={\left(S_{2}-S_{1}\right)}_{sys}$$

(20) $$(X_{Q,in}-X_{Q,out})+(X_{W,in}-X_{W,out})+(X_{mass,in}-X_{mass,out})-X_{destroyed}=\;{\left(X_{2}-X_{1}\right)}_{sys}$$

If the process between states 1 and 2 were *reversible*, there would be no exergy destruction (*X*_destroyed_ = 0) and the net useful work output (*W*_net,out_ = *X_W_*_,out_ − *X_W_*_,in_) in the exergy balance equation would be the reversible work output *W*_rev,out_. Then, the *reversible work output* for a process from state 1 to state 2 becomes the following:

(21) $$W_{rev,out}=\left(X_{Q,in}-X_{Q,out}\right)+\left(X_{mass,in}-X_{mass,out}\right)+{\left(X_{1}-X_{2}\right)}_{sys}$$

The reversible work output *W*_rev,out_ for a process represents the maximum useful work that can be performed during that process. For a system involving heat transfer with many heat reservoirs and fluid streams entering and exiting, the maximum useful work that can be performed is the *sum* of (1) the net exergy transfer into the system by heat transfer, (2) the net exergy transfer into the system by mass flow, and (3) the decrease in the exergy of the system. The *actual* useful work output for the process will be lower. The difference between the reversible work output and the actual useful work output is the exergy destroyed, *X*_destroyed_ = *W*_rev,out_ − *W*_act,out_, also called *lost work*.

A negative value for *W*_rev,out_ indicates *reversible work input* *W*_rev,in_, which is the minimum work input required for the process to take place. In this case, exergy destroyed is the difference between the actual useful work input and the reversible work input, *X*_destroyed_ = *W*_act,in_ − *W*_rev,in_.

For a *steady-flow* system, the general *exergy balance* and *reversible work* relations can be written in the *rate form* as follows:

(22) $$({\dot{X}}_{Q,\mathrm{in}}-\;\;{\dot{X}}_{Q,\mathrm{out}})+\;({\dot{X}}_{W,\mathrm{in}}-\;{\dot{X}}_{W,\mathrm{out}})\;+\;({\dot{X}}_{\mathrm{mass},\mathrm{in}}-\;{\dot{X}}_{\mathrm{mass},\mathrm{out}})-\;{\dot{X}}_{\mathrm{destroyed}}=0$$

(23) $${\dot{W}}_{\mathrm{rev},\mathrm{out}}=\left({\dot{X}}_{Q,in}-{\dot{X}}_{Q,out}\right)+\left({\dot{X}}_{\mathrm{mass},\mathrm{in}}-{\dot{X}}_{\mathrm{mass},\mathrm{out}}\right)$$

where overdots denote *time rate*, and

(24) $${\dot{X}}_{Q}=\left(1-T_{0}/T_{b}\right)\dot{Q}\;\mathrm{and}\;{\dot{X}}_{\mathrm{mass}}=\dot{m}x_{\mathrm{flow}}=\dot{m}[\left(h-h_{0}\right)-T_{0}\left(s-s_{0}\right)+\mathrm{ke}+\mathrm{pe}]$$

A negative value obtained for $\dot{W}$_rev,out_ indicates reversible power input. For a *single-stream* steady-flow device with a mass flow rate $\dot{m}$ entering at state 1 and exiting at state 2, Equation (23) becomes the following:

(25) $${\dot{W}}_{\mathrm{rev},\mathrm{out}}=\sum \left(1-\frac{T_{0}}{T_{b}}\right){\dot{Q}}_{\mathrm{in}}-\sum \left(1-\frac{T_{0}}{T_{b}}\right){\dot{Q}}_{\mathrm{out}}+\dot{m}[\left(h_{1}-h_{2}\right)-T_{0}\left(s_{1}-s_{2}\right)\;+\left({\mathrm{ke}}_{1}-{\mathrm{ke}}_{2}\right)+({\mathrm{pe}}_{1}-{\mathrm{pe}}_{2})]\;\;\;\;\;$$

For an *adiabatic* system, it reduces to the following:

(26) $${\dot{W}}_{\mathrm{rev},\mathrm{out},\mathrm{adiabatic}}=\dot{m}[\left(h_{1}-h_{2}\right)-T_{0}\left(s_{1}-s_{2}\right)+\left({\mathrm{ke}}_{1}-{\mathrm{ke}}_{2}\right)+({\mathrm{pe}}_{1}-{\mathrm{pe}}_{2})]$$

Entropy generation and exergy destruction for a general steady-flow system are given as follows:

(27) $${\dot{S}}_{\mathrm{gen}}=\Sigma \dot{m}\left(s_{\mathrm{exit}}-s_{\mathrm{inlet}}\right)-\Sigma \dot{Q}/T_{b}\;\;\mathrm{and}\;\;{\dot{X}}_{\mathrm{destroyed}}=T_{0}{\dot{S}}_{\mathrm{gen}}$$

For a one-inlet one-exit *adiabatic* steady-flow system, they reduce to the following:

(28) $${\dot{S}}_{\mathrm{gen},\mathrm{adiabatic}}=\dot{m}\left(s_{2}-s_{1}\right)\;\;\mathrm{and}\;\;{\dot{X}}_{\mathrm{destroyed},\mathrm{adiabatic}}=T_{0}{\dot{S}}_{\mathrm{gen}}=T_{0}\dot{m}(s_{2}-s_{1})$$

### 4.1. Exergy Expended–Recovered Formulation

In this approach, exergy loss associated with *heat loss* or *mass purged* to the environment is treated as part of the exergy recovered. This simplifies the analysis since the need to use the term *exergy loss* is alleviated. When exergy expended or consumed within a steady-flow system is taken as the base, exergy efficiency is expressed as follows:

(29) $$\eta_{\mathrm{ex}}=\frac{{\dot{X}}_{\mathrm{recovered}}}{{\dot{X}}_{\mathrm{expended}}}=1-\frac{{\dot{X}}_{\mathrm{destroyed}}}{{\dot{X}}_{\mathrm{expended}}}$$

The $\dot{X}$_expended_ and $\dot{X}$_recovered_ expressions depend on whether the exergy of the fluid stream increases or decreases as it passes through the device.

(*a*) $∆{\dot{X}}_{\mathrm{mass}}={\dot{X}}_{\mathrm{mass},\mathrm{out}}-{\dot{X}}_{\mathrm{mass},\mathrm{in}}={\dot{X}}_{2}-{\dot{X}}_{1}<0$: Exergy decrease in the fluid stream counts as exergy expended:

(30) $${\dot{X}}_{\mathrm{expended}}={\dot{X}}_{W,\mathrm{in}}+{\dot{X}}_{Q,\mathrm{in}}+({\dot{X}}_{1}-{\dot{X}}_{2})$$

(31) $${\dot{X}}_{\mathrm{recovered}}={\dot{X}}_{W,\mathrm{out}}+{\dot{X}}_{Q,\mathrm{out}}$$

(32) $$\eta_{\mathrm{ex}}=\frac{{\dot{X}}_{\mathrm{recovered}}}{{\dot{X}}_{\mathrm{expended}}}=\frac{{\dot{X}}_{W,\mathrm{out}}+{\dot{X}}_{Q,\mathrm{out}}}{{\dot{X}}_{W,\mathrm{in}}+{\dot{X}}_{Q,\mathrm{in}}+({\dot{X}}_{1}-{\dot{X}}_{2})}\;\;\;\;\;\;\;\;=1-\frac{{\dot{X}}_{\mathrm{destroyed}}}{{\dot{X}}_{W,\mathrm{in}}+{\dot{X}}_{Q,\mathrm{in}}+({\dot{X}}_{1}-{\dot{X}}_{2})}$$

(*b*) $∆{\dot{X}}_{\mathrm{mass}}={\dot{X}}_{\mathrm{mass},\mathrm{out}}-{\dot{X}}_{\mathrm{mass},\mathrm{in}}={\dot{X}}_{2}-{\dot{X}}_{1}>0$: Exergy increase in the fluid stream counts as exergy recovered:

(33) $${\dot{X}}_{\mathrm{expended}}={\dot{X}}_{W,\mathrm{in}}+{\dot{X}}_{Q,\mathrm{in}}$$

(34) $${\dot{X}}_{\mathrm{recovered}}={\dot{X}}_{W,\mathrm{out}}+{\dot{X}}_{Q,\mathrm{out}}+({\dot{X}}_{2}-{\dot{X}}_{1})$$

(35) $$\eta_{\mathrm{ex}}=\frac{{\dot{X}}_{\mathrm{recovered}}}{{\dot{X}}_{\mathrm{expended}}}=\frac{{\dot{X}}_{W,\mathrm{out}}+{\dot{X}}_{Q,\mathrm{out}}+({\dot{X}}_{2}-{\dot{X}}_{1})}{{\dot{X}}_{W,\mathrm{in}}+{\dot{X}}_{Q,\mathrm{in}}}=1-\frac{{\dot{X}}_{\mathrm{destroyed}}}{{\dot{X}}_{W,\mathrm{in}}+{\dot{X}}_{Q,\mathrm{in}}}$$

For adiabatic (${\dot{X}}_{Q,\mathrm{in}}={\dot{X}}_{Q,\mathrm{out}}=0$) work-producing (${\dot{X}}_{W,\mathrm{in}}=0$) devices such as turbines and work-consuming (${\dot{X}}_{W,\mathrm{out}}=0$) devices such as compressors, pumps, and fans, exergy efficiency relations reduce to the following:

(36) $$\eta_{\mathrm{ex},\mathrm{adiabatic},W\_\mathrm{out}}=\frac{{\dot{X}}_{\mathrm{recovered}}}{{\dot{X}}_{\mathrm{expended}}}=\frac{{\dot{X}}_{W,\mathrm{out}}}{{\dot{X}}_{1}-{\dot{X}}_{2}}=\frac{{\dot{W}}_{\mathrm{act},\mathrm{out}}}{{\dot{W}}_{\mathrm{rev},\mathrm{out}}}$$

(37) $$\eta_{\mathrm{ex},\mathrm{adiabatic},W\_\mathrm{in}}=\frac{{\dot{X}}_{\mathrm{recovered}}}{{\dot{X}}_{\mathrm{expended}}}=\frac{{\dot{X}}_{2}-{\dot{X}}_{1}}{{\dot{X}}_{W,\mathrm{in}}}=\frac{{\dot{W}}_{\mathrm{rev},\mathrm{in}}}{{\dot{W}}_{\mathrm{act},\mathrm{in}}}$$

which are identical to the widely used exergy efficiency relations *η*_ex_ = $\dot{W}$_act,out_/$\dot{W}$_rev,out_ for adiabatic turbines and *η*_ex_ = $\dot{W}$_rev,in_/$\dot{W}$_act,in_ for adiabatic compressors, pumps, and fans. Therefore, the exergy expended–recovered approach is quite suitable for determining the exergy efficiency of work-producing and work-consuming steady-flow devices such as turbines, compressors, and pumps.

The two exergy efficiency relations above for *adiabatic devices* are also applicable to the *extended system* (${\dot{X}}_{Q,\mathrm{loss}}=0$ and ${\dot{X}}_{\mathrm{mass},\mathrm{purged}}=0$) analysis of non-adiabatic work-producing or work-consuming devices, provided that *they involve heat transfer only with the environment* (and thus ${\dot{X}}_{Q,\mathrm{loss}}=0$). The exergy efficiency of the extended system will be *lower* than that of the physical system since $\eta_{ex}=1-{\dot{X}}_{\mathrm{destroyed}}/{\dot{X}}_{\mathrm{expended}}$, and the exergy destroyed term in the extended system case includes exergy destruction within the immediate surroundings associated with heat loss and the substances purged to the environment, if any.

### 4.2. Exergy Input–Output Formulation

In this approach, exergy losses associated with *heat loss* and *mass purged* to the environment are treated as *exergy loss*, and they are not included in the exergy output. That is, exergy transfer associated with heat loss to the environment is included in the exergy loss term ${\dot{X}}_{\mathrm{loss}}$ while exergy transfer associated with heat transfer to other systems ${\dot{X}}_{Q,\mathrm{out}}$ (as in cogeneration systems, for example) is included in the exergy output term ${\dot{X}}_{\mathrm{output}}$. The exergy of the mass exhausted to the environment (including its associated chemical exergy, if any) is also included in the *exergy loss* term ${\dot{X}}_{\mathrm{loss}}$, but not in the *exergy output* term.

When exergy input to the steady-flow system is taken as the base, exergy efficiency is expressed as follows:

(38) $$\eta_{\mathrm{ex}}=\frac{{\dot{X}}_{\mathrm{output}}}{{\dot{X}}_{\mathrm{input}}}=1-\frac{{\dot{X}}_{\mathrm{destroyed}}+{\dot{X}}_{\mathrm{loss}}}{{\dot{X}}_{\mathrm{input}}}$$

The $\dot{X}$_input_ and $\dot{X}$_output_ expressions depend on whether the exergy of the fluid stream increases or decreases as it passes through the system, and thus these two cases should be analyzed separately.

(*a*) $∆{\dot{X}}_{\mathrm{mass}}={\dot{X}}_{\mathrm{mass},\mathrm{out}}-{\dot{X}}_{\mathrm{mass},\mathrm{in}}={\dot{X}}_{2}-{\dot{X}}_{1}<0$: Exergy decrease in the fluid stream counts as exergy input:

(39) $${\dot{X}}_{\mathrm{input}}={\dot{X}}_{W,\mathrm{in}}+{\dot{X}}_{Q,\mathrm{in}}+({\dot{X}}_{1}-{\dot{X}}_{2})$$

(40) $${\dot{X}}_{\mathrm{output}}={\dot{X}}_{W,\mathrm{out}}+{\dot{X}}_{Q,\mathrm{out}}$$

(41) $${\dot{X}}_{\mathrm{loss}}={\dot{X}}_{Q,\mathrm{loss}}+{\dot{X}}_{\mathrm{mass},\mathrm{purged}}$$

(42) $$\eta_{\mathrm{ex}}=\frac{{\dot{X}}_{\mathrm{output}}}{{\dot{X}}_{\mathrm{input}}}=\frac{{\dot{X}}_{W,\mathrm{out}}+{\dot{X}}_{Q,\mathrm{out}}}{{\dot{X}}_{W,\mathrm{in}}+{\dot{X}}_{Q,\mathrm{in}}+({\dot{X}}_{1}-{\dot{X}}_{2})}=1-\frac{{\dot{X}}_{\mathrm{destroyed}}+{\dot{X}}_{\mathrm{loss}}}{{\dot{X}}_{W,\mathrm{in}}+{\dot{X}}_{Q,\mathrm{in}}+({\dot{X}}_{1}-{\dot{X}}_{2})}$$

Equation (42) resembles Equation (32) obtained by the exergy expended-recovered approach, but there are differences: Here the term ${\dot{X}}_{Q,\mathrm{out}}$ does not include ${\dot{X}}_{Q,\mathrm{loss}}$ associated with heat loss to the environment, and the term ${\dot{X}}_{\mathrm{loss}}$ is added to the exergy destruction term ${\dot{X}}_{\mathrm{destroyed}}$ in the last expression.

(*b*) $∆{\dot{X}}_{\mathrm{mass}}={\dot{X}}_{\mathrm{mass},\mathrm{out}}-{\dot{X}}_{\mathrm{mass},\mathrm{in}}={\dot{X}}_{2}-{\dot{X}}_{1}>0$: Exergy increase in the fluid stream counts as exergy output:

(43) $${\dot{X}}_{\mathrm{input}}={\dot{X}}_{W,\mathrm{in}}+{\dot{X}}_{Q,\mathrm{in}}$$

(44) $${\dot{X}}_{\mathrm{output}}={\dot{X}}_{W,\mathrm{out}}+{\dot{X}}_{Q,\mathrm{out}}+({\dot{X}}_{2}-{\dot{X}}_{1})$$

(45) $${\dot{X}}_{\mathrm{loss}}={\dot{X}}_{Q,\mathrm{loss}}+{\dot{X}}_{\mathrm{mass},\mathrm{purged}}$$

(46) $$\eta_{\mathrm{ex}}=\frac{{\dot{X}}_{\mathrm{output}}}{{\dot{X}}_{\mathrm{input}}}=\frac{{\dot{X}}_{W,\mathrm{out}}+{\dot{X}}_{Q,\mathrm{out}}+({\dot{X}}_{2}-{\dot{X}}_{1})}{{\dot{X}}_{W,\mathrm{in}}+{\dot{X}}_{Q,\mathrm{in}}}=1-\frac{{\dot{X}}_{\mathrm{destroyed}}+{\dot{X}}_{\mathrm{loss}}}{{\dot{X}}_{W,\mathrm{in}}+{\dot{X}}_{Q,\mathrm{in}}}$$

Equation (46) resembles Equation (35) obtained by the exergy expended-recovered approach, but there are differences: Here the term ${\dot{X}}_{Q,\mathrm{out}}$ does not include ${\dot{X}}_{Q,\mathrm{loss}}$ associated with heat loss to the environment, and the term ${\dot{X}}_{\mathrm{loss}}$ is added to the exergy destruction term ${\dot{X}}_{\mathrm{destroyed}}$ in the last expression. That is, the term included in the exergy loss relation ${\dot{X}}_{\mathrm{loss}}={\dot{X}}_{Q,\mathrm{loss}}+{\dot{X}}_{\mathrm{mass},\mathrm{purged}}$ should be excluded from the exergy output relation to avoid double-counting since, in the exergy input–output formulation, exergy output is intended to be the *useful* exergy output. For a system such as a natural gas-powered air compressor that involves chemical reactions, for example, the exergy associated with exhaust gases ${\dot{X}}_{\mathrm{mass},\mathrm{purged}}$ as well as heat loss to the environment, ${\dot{X}}_{Q,\mathrm{out}}$ should not be included in the evaluation of exergy output.

For adiabatic (${\dot{X}}_{Q,\mathrm{in}}={\dot{X}}_{Q,\mathrm{out}}=0$) work-producing (${\dot{X}}_{W,\mathrm{in}}=0$) devices such as turbines and work-consuming (${\dot{X}}_{W,\mathrm{out}}=0$) devices such as compressors, pumps, and fans, the exergy efficiency relations reduce to the following:

(47) $$\eta_{\mathrm{ex},\mathrm{adiabatic},W\_\mathrm{out}}=\frac{{\dot{X}}_{\mathrm{output}}}{{\dot{X}}_{\mathrm{input}}}=\frac{{\dot{X}}_{W,\mathrm{out}}}{{\dot{X}}_{1}-{\dot{X}}_{2}}=\frac{{\dot{W}}_{\mathrm{act},\mathrm{out}}}{{\dot{W}}_{\mathrm{rev},\mathrm{out}}}$$

(48) $$\eta_{\mathrm{ex},\mathrm{adiabatic},W\_\mathrm{in}}=\frac{{\dot{X}}_{\mathrm{output}}}{{\dot{X}}_{\mathrm{input}}}=\frac{{\dot{X}}_{2}-{\dot{X}}_{1}}{{\dot{X}}_{W,\mathrm{in}}}=\frac{{\dot{W}}_{\mathrm{rev},\mathrm{in}}}{{\dot{W}}_{\mathrm{act},\mathrm{in}}}$$

which are identical to the expressions obtained above with the exergy expended–recovered approach. Therefore, both approaches give the same result for exergy efficiency of adiabatic work-producing and work-consuming devices in steady operation.

This is also the case for *extended systems* since ${\dot{X}}_{\mathrm{loss}}={\dot{X}}_{Q,\mathrm{loss}}+{\dot{X}}_{\mathrm{mass},\mathrm{purged}}$ = 0 and the exergy efficiency relations of exergy input–output approach reduce to those obtained by the exergy expended–recovered approach:

(49) $$(a)\;\Delta {\dot{X}}_{\mathrm{mass}}\;<\;0:\;\eta_{\mathrm{ex},\mathrm{extended}}=\frac{{\dot{X}}_{\mathrm{output}}}{{\dot{X}}_{\mathrm{input}}}=\frac{{\dot{X}}_{W,\mathrm{out}}+{\dot{X}}_{Q,\mathrm{out}}}{{\dot{X}}_{W,\mathrm{in}}+{\dot{X}}_{Q,\mathrm{in}}+({\dot{X}}_{1}-{\dot{X}}_{2})}=1-\frac{{\dot{X}}_{\mathrm{destroyed}}}{{\dot{X}}_{W,\mathrm{in}}+{\dot{X}}_{Q,\mathrm{in}}+({\dot{X}}_{1}-{\dot{X}}_{2})}$$

(50) $$(b)\;\Delta {\dot{X}}_{\mathrm{mass}}\;>\;0:\;\eta_{\mathrm{ex},\mathrm{extended}}=\frac{{\dot{X}}_{W,\mathrm{out}}+{\dot{X}}_{Q,\mathrm{out}}+({\dot{X}}_{2}-{\dot{X}}_{1})}{{\dot{X}}_{W,\mathrm{in}}+{\dot{X}}_{Q,\mathrm{in}}}=1-\frac{{\dot{X}}_{\mathrm{destroyed}}}{{\dot{X}}_{W,\mathrm{in}}+{\dot{X}}_{Q,\mathrm{in}}}\;\;\;\;\;\;\;\;$$

Therefore, either approach can be used to determine the exergy efficiency of a general steady-flow system when the *extended system* is taken as the system instead of the physical system. This is not surprising since the denominators of the exergy efficiency relations of both approaches are identical, ${\dot{X}}_{\mathrm{expended}}={\dot{X}}_{\mathrm{input}}$. The numerators also appear the same, except that the term ${\dot{X}}_{Q,\mathrm{out}}$ includes the exergy loss term ${\dot{X}}_{Q,\mathrm{loss}}$ associated with heat loss to the environment in the exergy expended–recovered approach, but not in the exergy input–output approach. As a result, when there is heat loss to the environment, exergy efficiency evaluated with the exergy expended–recovered approach will be higher than that evaluated with the exergy input–output approach.

Both formulations presented above are plausible, and the appropriate choice depends on how the analyst prefers to treat the effects of heat loss to the environment and mass exhausted. A simpler and more realistic approach is to analyze the *extended system* for which both approaches give the same result for exergy efficiency. As expected, the exergy efficiency of the extended system will be lower since it reflects the irreversibilities that occur in the immediate surroundings as well as within the system. Overall, however, the exergy expended–recovered approach appears to be easier to work with since it does not require dealing with exergy loss. In addition, there is nothing wrong with calculating more than one exergy efficiency (one for the physical system and another for the extended system, for example) to gain more insights about the system’s performance.

### 4.3. Reversible Work Formulation

This formulation is meaningful for only work-producing or work-consuming steady-flow devices. When reversible work, which is the maximum work output that can be produced or the minimum work input required, is taken as the base, exergy efficiency can be expressed in terms of actual work and reversible work as follows:

Work-producing devices (turbines):

(51) $$\eta_{\mathrm{ex},W\_\mathrm{out}}=\frac{{\dot{W}}_{\mathrm{act},\mathrm{out}}}{{\dot{W}}_{\mathrm{rev},\mathrm{out}}}=\frac{{\dot{W}}_{\mathrm{act},\mathrm{out}}}{{\dot{X}}_{Q,\mathrm{in}}-{\dot{X}}_{Q,\mathrm{out}}+({\dot{X}}_{1}-{\dot{X}}_{2})}=\frac{{\dot{X}}_{W,\mathrm{out}}}{{\dot{X}}_{Q,\mathrm{in}}-{\dot{X}}_{Q,\mathrm{out}}+{\dot{(X}}_{1}-{\dot{X}}_{2})}$$

Work-consuming devices (compressors, pumps, fans):

(52) $$\eta_{\mathrm{ex},W\_\mathrm{in}}=\frac{{\dot{W}}_{\mathrm{rev},\mathrm{in}}}{{\dot{W}}_{\mathrm{act},\mathrm{in}}}=\frac{{\dot{X}}_{Q,\mathrm{out}}-{\dot{X}}_{Q,\mathrm{in}}+({\dot{X}}_{2}-{\dot{X}}_{1})}{{\dot{W}}_{\mathrm{act},\mathrm{in}}}=\frac{{\dot{X}}_{Q,\mathrm{out}}-{\dot{X}}_{Q,\mathrm{in}}+\left({\dot{X}}_{2}-{\dot{X}}_{1}\right)}{{\dot{X}}_{W,\mathrm{in}}}$$

For *adiabatic* (${\dot{X}}_{Q,\mathrm{in}}={\dot{X}}_{Q,\mathrm{out}}=0$) work-producing (${\dot{X}}_{W,\mathrm{in}}=0$) and work-consuming (${\dot{X}}_{W,\mathrm{out}}=0$) devices, exergy efficiency relations reduce to the following:

(53) $$\eta_{\mathrm{ex},\mathrm{adiabatic},W\_\mathrm{out}}=\frac{{\dot{W}}_{\mathrm{act},\mathrm{out}}}{{\dot{W}}_{\mathrm{rev},\mathrm{out}}}=\frac{{\dot{W}}_{\mathrm{act},\mathrm{out}}}{{\dot{X}}_{1}-{\dot{X}}_{2}}=\frac{{\dot{X}}_{W,\mathrm{out}}}{{\dot{X}}_{1}-{\dot{X}}_{2}}$$

(54) $$\eta_{\mathrm{ex},\mathrm{adiabatic},W\_\mathrm{in}}=\frac{{\dot{W}}_{\mathrm{rev},\mathrm{in}}}{{\dot{W}}_{\mathrm{act},\mathrm{in}}}=\frac{{\dot{X}}_{2}-{\dot{X}}_{1}}{{\dot{W}}_{\mathrm{act},\mathrm{in}}}=\frac{{\dot{X}}_{2}-{\dot{X}}_{1}}{{\dot{X}}_{W,\mathrm{in}}}$$

which are identical to the results obtained above for adiabatic work-producing and work-consuming devices using the exergy expended–recovered and exergy input–output approaches.

### 4.4. Mixtures and Chemical Reactions

When a process involves concentration changes in fluid streams via mixing, separation, or chemical reactions, exergy calculations require the use of the property *chemical potential μ*, which is the *differential change in the total Gibbs function* of a mixture in a specified phase per differential change in a component in the same phase at mixture *P* and *T* while the mole numbers of all other components are held constant. A mixture is said to be an *ideal mixture* or an *ideal solution* when the effect of dissimilar molecules on each other in a gas or liquid mixture is negligible. Many liquid solutions encountered in practice, especially diluted ones, closely satisfy this condition and can be treated as ideal solutions with negligible error.

The *specific Gibbs function* (or *Gibbs free energy*) of a pure substance is defined as the combination property *g* = *h* − *Ts*. The chemical potential *μ* of a *pure substance* in a given phase is equivalent to its Gibbs function, $g_{\mathrm{pure}\;\mathrm{substance}}=g=h-Ts$. Therefore, the difference between the chemical potential and the Gibbs function in a mixture is due to the effect of *dissimilar molecules* in the mixture on each other. For an ideal mixture or solution, the *chemical potential* of component *i* is given as follows [1]:

(55) $${\bar{\mu }}_{i}={\bar{g}}_{i}={\bar{h}}_{i}-T{\bar{s}}_{i}$$

where *overbar* denotes properties per unit mole. When the temperature *T* of the pure substance equals the environment temperature *T*_0_, *chemical exergy* is expressed as follows:

*x*_ch,pure,@*T*=*T*_0_ = *x*_flow,pure_ = [(*h − T*_0_*s*) − (*h*_0_ − *T*_0_*s*_0_)]_@*T*_0_ = [*g* − *g*_0_]_@*T*_0_ = [*μ* − *μ*_0_]_@*T*_0_ (56)

Therefore, the *chemical exergy* of a flowing fluid at *T*_0_ is equal to the difference between the *Gibbs function* (or between the *chemical potential*) of the system at the given pressure *P* and *T*_0_ and at *P*_0_ and *T*_0_. For a *component i* in an ideal mixture or ideal solution, *chemical exergy* is expressed as follows:

*x*_ch,*i*,*T*=*T*_0_ = *x*_flow,*i*_ = [(*h_i_ − T*_0_*s_i_*) − (*h_i_*_,0_ − *T*_0_*s_i_*_,0_)]_*T*___0_ = [*g_i_* − *g_i_*_,0_]_@*T*_0_ = [*μ_i_* − *μ_i_*_,0_]_@*T*_0_ (57)

The chemical exergy of the gas mixture per unit mole of mixture at dead state temperature *T*_0_ and pressure *P*_0_ is expressed in terms of *pure substance standard chemical exergies of components* as follows:

(58) $${\bar{x}}_{\mathrm{ch}.\mathrm{mixture}}=\sum y_{i}{\bar{x}}_{\mathrm{ch},i}^{0}+R_{u}T_{0}\sum y_{i}lny_{i}$$

where *y_i_* = *N_i_*/*N_m_* is the *mole fraction* of component *i*, *N_i_* is the mole number of component *i*, *N_m_* = ∑*N_i_* is the total number of moles of the mixture, ${\bar{x}}_{\mathrm{ch}}^{0}$ is the *standard chemical exergy* of a pure substance at *T*_0_ and *P*_0_, and *R_u_* = 8.314 kJ/kmol·K is the universal gas constant. The superscript 0 denotes the standard reference conditions of *T*_0_ and *P*_0_. The total chemical exergy of an ideal mixture or solution is expressed as follows:

(59) $$X_{\mathrm{ch}.\mathrm{mixture}}=N_{m}{\bar{x}}_{\mathrm{ch}.\mathrm{mixture}}=\sum N_{i}{\bar{x}}_{\mathrm{ch},i}=\sum N_{i}{\bar{x}}_{\mathrm{ch},i}^{0}+R_{u}T_{0}\sum N_{i}lny_{i}$$

This represents the work that can be produced if the mixture is allowed to mix with the environment in a reversible manner until each component reaches the concentration level in the environment and thus achieves chemical equilibrium with the environment. The *standard environment* in the gas phase is typically represented by the atmospheric air at *T*_0_ = 25 °C and *P*_0_ = 1 atm with fixed mole fractions of *y_i_*_,env_.

Exergy is a measure of work potential, and thus the chemical exergy relations given above are also relations for reversible work. Therefore, assuming ideal mixtures or ideal solutions, the *maximum possible work* that can be obtained from a mixture at *T*_0_ and *P*_0_ as it reaches chemical equilibrium with the environment is as follows:

(60) $$W_{\mathrm{rev},\mathrm{out},\mathrm{mixing}}=\sum N_{i}{\bar{x}}_{\mathrm{ch},i}^{0}+R_{u}T_{0}\sum N_{i}lny_{i}$$

The chemical exergy relations given above are limited to systems that are at temperature *T*_0_ and pressure *P*_0_ of the environment and involve no chemical reactions. Considering that the exergy of a substance becomes equal to its Gibbs function when *T* = *T*_0_, the exergy associated with the formation of a substance is simply the *Gibbs function of formation* of that substance at the standard reference state, ${\bar{x}}_{i,\mathrm{formation}}={\bar{g}}_{f,i}^{0}$. Therefore, when chemical reactions are involved, chemical exergy of a component *i* is obtained by adding the exergy of formation to the chemical exergy relations given above:

(61) $${\bar{x}}_{\mathrm{ch}.\mathrm{mixture}}=\sum y_{i}{\bar{g}}_{f,i}^{0}+\sum y_{i}{\bar{x}}_{\mathrm{ch},i}=\sum y_{i}{\bar{g}}_{f,i}^{0}+\sum y_{i}{\bar{x}}_{\mathrm{ch},i}^{0}+R_{u}T_{0}\sum y_{i}lny_{i}$$

(62) $$X_{\mathrm{ch}.\mathrm{mixture}}=N_{m}{\bar{x}}_{\mathrm{ch}.\mathrm{mixture}}=\sum N_{i}{\bar{x}}_{\mathrm{ch},i}\;\;\;\;\;\;\;\;\;\;\;\;\;\;=\sum N_{i}{\bar{g}}_{f,i}^{0}+\sum N_{i}{\bar{x}}_{\mathrm{ch},i}^{0}+R_{u}T_{0}\sum N_{i}lny_{i}$$

If a mixture enters a system at *T*_1_ and *P*_1_ instead of *T*_0_ and *P*_0_ and the kinetic and potential energies are to be considered, the exergy change ${\bar{X}}_{1}-{\bar{X}}_{0}=\sum N_{i}{\left[\left({\bar{h}}_{1}-{\bar{h}}_{0}\right)-T_{0}\left({\bar{s}}_{1}-{\bar{s}}_{0}\right)+{\bar{\mathrm{ke}}}_{1}+{\bar{\mathrm{pe}}}_{1}\right]}_{i}$ associated with this change in state should be added to the exergy of the mixture. For a reaction chamber, the exergy change associated with the chemical reaction can be determined by writing the mixture exergy relation for the reactants and the products and taking their difference.

## 5. Exergy Efficiency of Steady-Flow Devices

General exergy efficiency relations for steady-flow systems are developed below using three different approaches. But first, we need to clarify how exergy flow associated with a fluid stream will be treated. One approach is to treat exergy flowing in with mass ${\dot{X}}_{\mathrm{mass},\mathrm{in}}=\dot{m}x_{\mathrm{flow},\mathrm{in}}$ as exergy input (or exergy expended), and exergy flowing out with mass ${\dot{X}}_{\mathrm{mass},\mathrm{out}}=\dot{m}x_{\mathrm{flow},\mathrm{out}}$ as exergy output (or exergy recovered). However, an energy system such as a power plant or refrigerator involves various components, and a fluid stream leaving one component enters another. As such, exergy supplied by a fluid stream to a component is the difference between the inlet and exit exergies, $∆{\dot{X}}_{\mathrm{mass}}={\dot{X}}_{\mathrm{mass},\mathrm{in}}-{\dot{X}}_{\mathrm{mass},\mathrm{out}}$ and it makes sense to treat the difference as the exergy input (or exergy expended).

If the fluid gains exergy as it flows through the device, then the difference $∆{\dot{X}}_{\mathrm{mass}}={\dot{X}}_{\mathrm{mass},\mathrm{out}}-{\dot{X}}_{\mathrm{mass},\mathrm{in}}$ is to be accounted as exergy output (or exergy recovered). Therefore, in the formulation below, we usually consider the exergy change in a fluid stream $∆{\dot{X}}_{\mathrm{mass}}$ as exergy input or output, depending on whether the exergy of the fluid stream is increasing or decreasing, rather than taking the exergy of the fluid entering as input and the exergy of the fluid exiting as output. This way, the fluid exit exergy ${\dot{X}}_{\mathrm{mass},\mathrm{out}}$ of one device becomes the fluid inlet exergy ${\dot{X}}_{\mathrm{mass},\mathrm{in}}$ of the next device. For single device systems, the quantities ${\dot{X}}_{\mathrm{mass},\mathrm{in}}$ and ${\dot{X}}_{\mathrm{mass},\mathrm{out}}$ can be treated as exergy input and output, respectively, if it is more meaningful and provides the best insight for the performance of the device.

Next, we develop specific exergy efficiency relations for devices that typically operate under steady conditions. We perform this by applying the general steady-flow exergy efficiency relations above to specific steady-flow devices such as turbines, compressors, pumps, nozzles, diffusers, valves, and heat exchangers. We use the *exergy expended–recovered approach* in the analysis but *reinterpret* what constitutes *exergy expended* and *exergy recovered* if necessary by considering the operation of the analyzed system and its intended outcome such that exergy efficiency provides the most insight into the operation. We will provide alternative definitions, if necessary, to develop different perspectives. Throughout the analysis, we assume the environmental conditions and thus the limited dead state is *T*_0_ = 25 °C and *P*_0_ = 100 kPa. Calculations are performed using EES software (v10) with built-in thermodynamic properties [58].

### 5.1. Turbines

The exergy efficiency of a turbine is determined by simplifying the general relation above for exergy expended–recovered approach for the case of Δ$\dot{X}$_mass_ < 0 (exergy of the fluid stream decreasing). The exergy decrease, $\dot{X}$_1_ − $\dot{X}$_2_, constitutes exergy expended. Noting that a turbine does not involve any heat or work input (it may involve heat loss) and thus $\dot{X}$*_W_*__in_ = 0 and $\dot{X}$*_Q_*__in_ = 0, exergy efficiency of a turbine can be expressed as follows:

(63) $${\dot{X}}_{\mathrm{expended}}\;={\dot{X}}_{\mathrm{mass},\mathrm{in}}-{\dot{X}}_{\mathrm{mass},\mathrm{out}}=\;{\dot{X}}_{1}\;-\;{\dot{X}}_{2}$$

(64) $${\dot{X}}_{\mathrm{recovered}}={\dot{X}}_{W\_\mathrm{out}}+{\dot{X}}_{Q\_\mathrm{out}}$$

(65) $$\eta_{\mathrm{ex}}=\frac{{\dot{X}}_{\mathrm{recovered}}}{{\dot{X}}_{\mathrm{expended}}}=\frac{{\dot{X}}_{W,\mathrm{out}}+{\dot{X}}_{Q,\mathrm{out}}}{{\dot{X}}_{1}-{\dot{X}}_{2}}=\frac{{\dot{W}}_{\mathrm{act},\mathrm{out}}+{\dot{X}}_{Q\_\mathrm{out}}}{{\dot{X}}_{1}-{\dot{X}}_{2}}$$

No attempt is made in practice to utilize the exergy loss $\dot{X}$*_Q_*__out_ associated with heat loss from the turbine. Therefore, it is convenient to consider an *extended system* that includes the immediate surroundings of the device so that the system boundaries are at environment temperature *T*_0_. Exergy loss associated with heat loss becomes zero in this case ($\dot{X}$*_Q_*_, out_ = 0) and exergy efficiency relation above simplifies to the following equation:

(66) $$\eta_{\mathrm{ex},\mathrm{extended}}=\frac{{\dot{X}}_{\mathrm{recovered}}}{{\dot{X}}_{\mathrm{expended}}}=\frac{{\dot{X}}_{W\_\mathrm{out}}}{{\dot{X}}_{1}-{\dot{X}}_{2}}=\frac{{\dot{W}}_{\mathrm{act},\mathrm{out}}}{{\dot{X}}_{1}-{\dot{X}}_{2}}$$

This relation is also valid for *adiabatic* turbines since $\dot{X}$*_Q_*__out_ = 0 when heat loss is zero.

The stead-flow reversible work relation reduces to $\dot{W}$_rev,out_ = $\dot{X}$_1_ − $\dot{X}$_2_ for an adiabatic turbine. Therefore, the exergy efficiency of a turbine can be expressed in terms of reversible work as follows:

(67) $$\eta_{\mathrm{ex},\mathrm{adibatic}}=\frac{{\dot{W}}_{\mathrm{act},\mathrm{out}}}{{\dot{W}}_{rev,out}}$$

This relation is also valid for the *extended* system when the turbine experiences heat loss, but the result in this case will reflect the inclusion of the exergy destruction within the immediate surroundings of the turbine.

Exergy efficiency of a turbine can also be determined from the following equation:

(68) $$\eta_{\mathrm{ex}}=1-\frac{{\dot{X}}_{\mathrm{destroyed}}}{{\dot{X}}_{\mathrm{expended}}}$$

where ${\dot{S}}_{\mathrm{gen}}=\dot{m}\left(s_{2}-s_{1}\right)+{\dot{Q}}_{\mathrm{out}}/T_{0}$ and ${\dot{X}}_{\mathrm{destroyed}}=T_{0}{\dot{S}}_{\mathrm{gen}}$. 

When kinetic and potential energy changes are negligible, the actual power output of a turbine (adiabatic or not) and reversible work can be determined in terms of fluid properties from the following equations:

(69) $${\dot{W}}_{\mathrm{act},\mathrm{out}}=\dot{m}\left(h_{1}-h_{2}\right)-{\dot{Q}}_{\mathrm{out}}$$

(70) $${\dot{W}}_{\mathrm{rev},\mathrm{out}}={\dot{X}}_{1}-{\dot{X}}_{2}=\dot{m}\left[\left(h_{1}-h_{2}\right)-T_{0}\left(s_{1}-s_{2}\right)\right]={\dot{X}}_{\mathrm{expended}}$$

**Figure 3:**
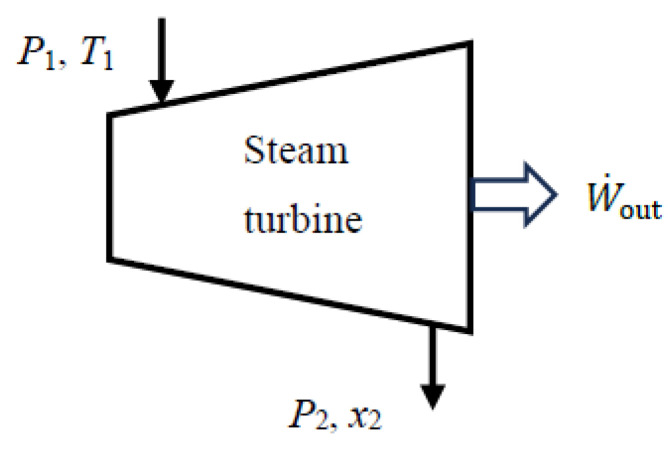
An adiabatic steam turbine.

As a numerical application, we calculate the exergy efficiency of an adiabatic steam turbine with a mass flow rate of $\dot{m}=0.5\;\mathrm{kg}/s$, inlet conditions of *P*_1_ = 3 MPa and *T*_1_ = 400 °C, exit conditions of *P*_2_ = 10 kPa and quality *x*_2_ = 0.90, and a power output of 444 kW (Figure 3).

We take the turbine in steady operation as the system. Using the steady-flow exergy efficiency relations given above, we obtain $\dot{X}$_expended_ = Δ$\dot{X}$_mass_ = 514 kW, $\dot{X}$_recovered_ = $\dot{X}$*_W_*__out_ = $\dot{W}$_act,out_ = 444 kW, and *η*_ex_ = $\dot{X}$_recovered_/$\dot{X}$_expended_ = 0.862. Exergy destroyed during this process is $\dot{X}$_destroyed_ = *T*_0_$\dot{S}$_gen_ = 70.8 kW, and the relation *η*_ex_ = 1 − $\dot{X}$_destroyed_/$\dot{X}$_expended_ gives the same result for exergy efficiency. The reversible work output during this process is $\dot{W}$_rev,out_ = 514 kW, and the relation *η*_ex_ = $\dot{W}$_act,out_/$\dot{W}$_rev,out_ also gives the same result, *η*_ex_ = 444/514 = 0.862. Therefore, 86.2% of the exergy consumed from the steam as it flows through the turbine is recovered as shaft power during this process while the remaining 13.8% is destroyed due to irreversibilities such as friction within the turbine.

### 5.2. Compressors

The exergy efficiency of a compressor (or a pump or fan) is determined by simplifying the general relation above obtained with the exergy expended–recovered approach for the case of Δ$\dot{X}$_mass_ > 0 (exergy of the fluid stream increasing). The exergy increase in the compressed gas, $\dot{X}$_2_ − $\dot{X}$_1_, is the exergy recovered. Noting that a compressor does not involve any work output and heat input (it may involve heat loss) and thus $\dot{X}$*_W_*__out_ = 0 and $\dot{X}$*_Q_*__in_ = 0, exergy efficiency of a compressor can be expressed as follows:

(71) $${\dot{X}}_{\mathrm{expended}}\;=\;{\dot{X}}_{W\_\mathrm{in}}\;=\;{\dot{W}}_{\mathrm{act},\mathrm{in}}$$

(72) $${\dot{X}}_{\mathrm{recovered}}={\dot{X}}_{\mathrm{mass},\mathrm{out}}-{\dot{X}}_{\mathrm{mass},\mathrm{in}}+{\dot{X}}_{Q\_\mathrm{out}}={\dot{X}}_{2}\;-\;{\dot{X}}_{1}+{\dot{X}}_{Q\_\mathrm{out}}$$

(73) $$\eta_{\mathrm{ex}}=\frac{{\dot{X}}_{\mathrm{recovered}}}{{\dot{X}}_{\mathrm{expended}}}=\frac{{\dot{X}}_{2}-{\dot{X}}_{1}+{\dot{X}}_{Q\_\mathrm{out}}}{{\dot{X}}_{W\_\mathrm{in}}}=\frac{{\dot{X}}_{2}-{\dot{X}}_{1}+{\dot{X}}_{Q\_\mathrm{out}}}{{\dot{W}}_{\mathrm{act},\;\mathrm{in}}}$$

No attempt is made in practice to utilize the exergy loss $\dot{X}$*_Q_*__out_ associated with cooling or heat loss from the compressor. Therefore, it is convenient to consider an *extended system* that includes the immediate surroundings of the device so that the system boundaries are at environment temperature *T*_0_. Exergy loss associated with heat loss becomes zero in this case ($\dot{X}$*_Q_*__out_ = 0) and exergy efficiency relation is simplified to the following equation:

(74) $$\eta_{\mathrm{ex},\mathrm{extended}}=\frac{{\dot{X}}_{\mathrm{recovered}}}{{\dot{X}}_{\mathrm{expended}}}=\frac{{\dot{X}}_{2}-{\dot{X}}_{1}}{{\dot{X}}_{W\_\mathrm{in}}}=\frac{{\dot{X}}_{2}-{\dot{X}}_{1}}{{\dot{W}}_{\mathrm{act},\mathrm{in}}}$$

(75) $$\eta_{\mathrm{ex}}=\frac{{\dot{W}}_{\mathrm{rev},\mathrm{in}}}{{\dot{W}}_{\mathrm{act},\mathrm{in}}}=\frac{{\dot{X}}_{Q\_\mathrm{out}}-{\dot{X}}_{Q\_\mathrm{in}}+\left({\dot{X}}_{2}-{\dot{X}}_{1}\right)}{{\dot{W}}_{\mathrm{act},\mathrm{in}}}=\frac{{\dot{X}}_{Q\_\mathrm{out}}-{\dot{X}}_{Q\_\mathrm{in}}+\left({\dot{X}}_{2}-{\dot{X}}_{1}\right)}{{\dot{X}}_{W\_\mathrm{in}}}$$

This relation is also valid for *adiabatic* compressors since $\dot{X}$*_Q_*__out_ = 0 when heat loss is zero. The steady-flow reversible work relation $\dot{W}$_rev,in_ = $\dot{X}$_2_ − $\dot{X}$_1_ + ($\dot{X}$*_Q_*__out_ − $\dot{X}$*_Q_*__in_) reduces to $\dot{W}$_rev,in_ = $\dot{X}$_2_ − $\dot{X}$_1_ for a compressor that involves no heat input or heat loss (adiabatic compressor). Therefore, the exergy efficiency of a compressor can be expressed in terms of reversible work as follows:

(76) $$\eta_{\mathrm{ex}}=\frac{{\dot{W}}_{\mathrm{rev},\mathrm{in}}}{{\dot{W}}_{act,in}}=\frac{{\dot{X}}_{2}-{\dot{X}}_{1}}{{\dot{W}}_{\mathrm{act},\mathrm{in}}}$$

This relation is also valid for the *extended* system when the compressor is cooled, but the result in this case will reflect the inclusion of the exergy destruction associated with heat loss during cooling.

Exergy efficiency can also be determined from the following equation:

(77) $$\eta_{\mathrm{ex}}=1-\frac{{\dot{X}}_{\mathrm{destroyed}}}{{\dot{X}}_{\mathrm{expended}}}$$

where ${\dot{S}}_{\mathrm{gen}}=\dot{m}\left(s_{2}-s_{1}\right)+{\dot{Q}}_{\mathrm{out}}/T_{0}$ and ${\dot{X}}_{\mathrm{destroyed}}=T_{0}{\dot{S}}_{\mathrm{gen}}$.

When kinetic and potential energy changes are negligible, the actual work input of a compressor (adiabatic or not) and reversible work can be determined in terms of fluid properties from the following equations:

(78) $${\dot{W}}_{\mathrm{act},\mathrm{in}}=\dot{m}\left(h_{2}-h_{1}\right)+{\dot{Q}}_{\mathrm{out}}$$

(79) $${\dot{W}}_{\mathrm{rev},\mathrm{in}}={\dot{X}}_{2}-{\dot{X}}_{1}=\dot{m}\left[\left(h_{2}-h_{1}\right)-T_{0}\left(s_{2}-s_{1}\right)\right]={\dot{X}}_{\mathrm{recovered}}$$

The relations given above for compressors are also valid for pumps and fans.

**Figure 4:**
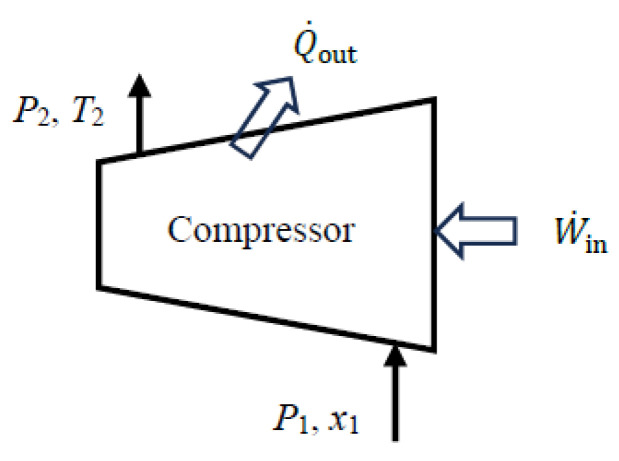
A refrigerant-134a compressor with heat loss.

As a numerical application, we calculate the exergy efficiency of a refrigerant-134a compressor with a mass flow rate of $\dot{m}=0.5\;\mathrm{kg}/s$, inlet conditions of *P*_1_ = 140 kPa and *x*_1_ = 1 (saturated vapor), exit conditions of *P*_2_ = 900 kPa and *T*_2_ = 50 °C, and a heat loss rate of 3.3 kW (Figure 4).

We consider the *extended system* that includes the compressor and its immediate surroundings. Using the steady-flow exergy efficiency relations given above, we obtain $\dot{X}$_expended_ = $\dot{X}$*_W_*__in_ = $\dot{W}$_act,in_ = 26.1 kW, $\dot{X}$_recovered_ = Δ$\dot{X}$_mass_ = 19.6 kW, and *η*_ex_ = $\dot{X}$_recovered_/$\dot{X}$_expended_ = 0.751. The exergy destroyed during this process is $\dot{X}$_destroyed_ = *T*_0_$\dot{S}$_gen_ = 6.50 kW, and the relation *η*_ex_ = 1 − $\dot{X}$_destroyed_/$\dot{X}$_expended_ also gives the same result for exergy efficiency. The reversible work input during this process is $\dot{W}$_rev,in_ = 19.6 kW, and the relation *η*_ex_ = $\dot{W}$_rev,in_/$\dot{W}$_act,in_ also gives the same result: *η*_ex_ = 19.6/26.1 = 0.751. Therefore, 75.1% of the exergy consumed by the compressor is recovered as an increase in the exergy of the refrigerant during this process while the remaining 24.9% is destroyed due to irreversibilities such as friction within the compressor and heat loss to the surroundings.

The total entropy generation associated with this process is $\dot{S}$_gen_= 0.0218 kW/K. Of this, 0.0210 kW/K occurs within the compressor and the remaining 0.0008 kW/K occurs within the immediate surroundings. The exergy destroyed within the compressor is $\dot{X}$_destroyed,comp_ = *T*_0_$\dot{S}$_gen,comp_ = 6.24 kW and the exergy efficiency of the compressor is *η*_ex,comp_ = 1 − $\dot{X}$_destroyed,comp_/$\dot{X}$_expended_ = 0.761. Therefore, accounting for the exergy destruction within the immediate surroundings of the compressor reduces exergy efficiency from 76.1% to 75.1%. If no effort is made to utilize the heat at 50 °C lost to the surroundings, the exergy efficiency of 75.1% for the extended system is a more realistic measure of performance than the 76.1% for the compressor alone.

### 5.3. Flow Through Valves

**Figure 5:**
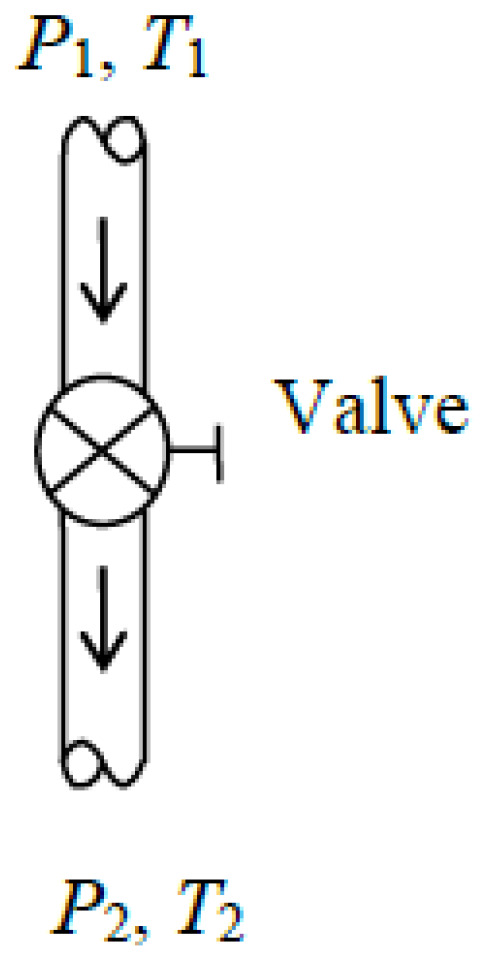
Schematic of a valve.

Valves are commonly used in flow systems to regulate the flow rate by restricting the flow and thus causing a pressure loss (Figure 5). In refrigeration systems, throttling valves or capillary tubes are used to inflict a large pressure loss, resulting in a significant decrease in refrigerant temperature. When the kinetic and potential energy changes in the fluid stream and heat transfer to or from the fluid are negligible, the energy balance equation for valves simplifies to *h*_2_ = *h*_1_.

Some exergy is always destroyed since valves generate entropy by introducing extra friction. Thus, there is no such thing as reversible valves to serve as idealized valves. A reversible turbine operating between the same pressure limits is also not a suitable model for a valve since the enthalpy of the fluid drops considerably in turbines instead of remaining constant. A turbine whose shaft is broken and thus provides no shaft work behaves like a valve because its internal components offer resistance to flow.

The decrease in the exergy of the fluid as it flows through the valve constitutes *exergy expended*. It is the difference between the inlet and exit exergies of the valve, as in the following equation:

(80) $${\dot{X}}_{\mathrm{expended}}={\dot{X}}_{\mathrm{mass},\mathrm{in}}-{\dot{X}}_{\mathrm{mass},\mathrm{out}}={\dot{X}}_{1}-{\dot{X}}_{2}=\dot{m}[(h_{1}-h_{2})-T_{0}(s_{1}-s_{2})]\;=\dot{m}T_{0}(s_{2}-s_{1})\;\;\;\;\;\;\;\;$$

since *h*_2_ = *h*_1_. Noting that entropy generation for a valve is ${\dot{S}}_{\mathrm{gen}}=\dot{m}(s_{2}-s_{1})$, exergy destroyed is as follows:

(81) $${\dot{X}}_{\mathrm{destroyed}}=T_{0}{\dot{S}}_{\mathrm{gen}}=T_{0}\dot{m}\left(s_{2}-s_{1}\right)={\dot{X}}_{\mathrm{expended}}$$

Then, it follows that exergy recovered is zero since ${\dot{X}}_{\mathrm{recovered}}={\dot{X}}_{\mathrm{expended}}-{\dot{X}}_{\mathrm{destroyed}}=0$. That is, in a valve, all of the expended exergy is destroyed and none of this exergy is recovered, ${\dot{X}}_{\mathrm{recovered}}=0$. Consequently, the exergy efficiency of a valve based on the exergy expended–recovered approach is always zero:

(82) $$\eta_{\mathrm{ex}}=\frac{{\dot{X}}_{\mathrm{recovered}}}{{\dot{X}}_{\mathrm{expended}}}=\frac{0}{{\dot{X}}_{1}-{\dot{X}}_{2}}=0\;\mathrm{or}\;\eta_{\mathrm{ex}}=1-\frac{{\dot{X}}_{\mathrm{destroyed}}}{{\dot{X}}_{\mathrm{expended}}}=1-\frac{{\dot{X}}_{1}-{\dot{X}}_{2}}{{\dot{X}}_{1}-{\dot{X}}_{2}}=0$$

This is expected since the entire exergy expended is wasted in the valve and thus ${\dot{X}}_{\mathrm{destroyed}}={\dot{X}}_{1}-{\dot{X}}_{2}$. Therefore, the proper exergy expended–recovered approach always gives zero for exergy efficiency of a valve regardless of the exergy value at the valve exit.

To remedy this deficiency and offer an alternative, we can take the exergy value at the valve inlet as the exergy expended and at the valve exit as the exergy recovered, and define exergy efficiency as their ratio as follows:

(83) $$\eta_{\mathrm{ex},\mathrm{alt}}=\frac{{\dot{X}}_{\mathrm{mass},\mathrm{out}}}{{\dot{X}}_{\mathrm{mass},\mathrm{in}}}=\frac{{\dot{X}}_{2}}{{\dot{X}}_{1}}=1-\frac{{\dot{X}}_{\mathrm{destroyed}}}{{\dot{X}}_{1}}$$

where

(84) $${\dot{X}}_{\mathrm{mass},\mathrm{in}}={\dot{X}}_{1}=\dot{m}[(h_{1}-h_{0})-T_{0}(s_{1}-s_{0})]$$

(85) $${\dot{X}}_{\mathrm{mass},\mathrm{out}}={\dot{X}}_{2}=\dot{m}[(h_{2}-h_{0})-T_{0}(s_{2}-s_{0})]$$

(86) $${\dot{X}}_{\mathrm{destroyed}}=T_{0}{\dot{S}}_{\mathrm{gen}}=T_{0}\dot{m}(s_{2}-s_{1})$$

The exergy expended–recovered approach categorically labels the valves as *exergy destroyers*, even though they fully *conserve energy*, regardless of the degree of destruction of the incoming exergy. The alternative approach, on the other hand, accounts for the recovered exergy at the valve exit, and calculates the exergy efficiency accordingly. Therefore, we recommend the use of Equation (83) for calculating exergy efficiency of the valves. The alternative definition makes it possible to get a *non-zero value* for the exergy efficiency of valves, making it possible to rate the valves by the fraction of the incoming exergy recovered at the valve exit. A high value of exergy efficiency in this case indicates that a small fraction of the incoming exergy is destroyed as it flows through the valve. For example, if the exergy of the fluid decreases from 100 kJ/kg at the valve inlet to 90 kJ/kg at the exit, the exergy efficiency of the valve becomes *η*_ex_ $={\dot{X}}_{\mathrm{mass},\mathrm{out}}/{\dot{X}}_{\mathrm{mass},\mathrm{in}}=90/100=$ 0.90 = 90%. When the exit exergy drops to 40 kJ/kg, exergy efficiency drops to 40 percent. Therefore, the alternative approach appears to be more suitable for calculating the exergy efficiency of valves.

As a numerical application, we calculate the exergy efficiency of a refrigerant-134a valve with a mass flow rate of $\dot{m}=0.5\;\mathrm{kg}/s$, inlet conditions of *P*_1_ = 800 kPa and *x*_1_ = 0 (saturated liquid), and exit conditions of *P*_2_ = 120 kPa (and *h*_2_ = *h*_1_).

We take the valve in steady operation as the system. Using the alternative exergy efficiency relation, we obtain ${\dot{X}}_{\mathrm{mass},\mathrm{in}}=21.59\;\mathrm{kW}$, ${\dot{X}}_{\mathrm{mass},\mathrm{out}}=17.17\;\mathrm{kW}$, and *η*_ex,alt_ $={\dot{X}}_{\mathrm{mass},\mathrm{out}}/{\dot{X}}_{\mathrm{mass},\mathrm{in}}=0.795$. Using the value of exergy destroyed gives the same result: ${\dot{X}}_{\mathrm{destroyed}}=4.42\;\mathrm{kW}$ and *η*_ex,alt_ $=1-{\dot{X}}_{\mathrm{destroyed}}/{\dot{X}}_{\mathrm{mass},\mathrm{in}}=0.795$. That is, 79.5% of the exergy content of the incoming refrigerant is retained at the valve exit in this case while the remaining 20.5% is destroyed within the valve. If the exit pressure were 600 kPa, the exergy efficiency would be 99%.

### 5.4. Flow Through Pipes and Ducts

Capillary tubes are often used in place of valves to cause a large pressure drop. Thus, the exergy efficiency of capillary tubes can be determined by using the relations given above for valves. In fact, flows through pipes and ducts always experience some pressure loss, and they can be treated as valves. The relation *η*_ex_ $={\dot{X}}_{\mathrm{mass},\mathrm{out}}/{\dot{X}}_{\mathrm{mass},\mathrm{in}}=1-{\dot{X}}_{\mathrm{destroyed}}/{\dot{X}}_{\mathrm{mass},\mathrm{in}}$ can be used to calculate the exergy efficiency of pipe or duct flow since those relations properly account for the effects of friction. For an *extended system* that includes the pipe or duct and its immediate surroundings, *T_b_* = *T*_0_ and thus ${\dot{X}}_{Q\_\mathrm{loss}}=0$. For a well-insulated pipe or duct, ${\dot{Q}}_{\mathrm{loss}}=0$ and thus ${\dot{S}}_{\mathrm{gen}}=\dot{m}(s_{2}-s_{1})$.

### 5.5. Nozzles

The purpose of a nozzle is to *increase the velocity*, and thus the *kinetic energy* of a fluid. Therefore, the change in the kinetic energy of the fluid should be included in nozzle analysis. Being a form of mechanical energy, the exergy associated with kinetic energy is the kinetic energy itself, *x*_ke_ = ke. A typical nozzle does not involve work or heat transfer other than heat loss to the environment ${\dot{Q}}_{\mathrm{loss}}$. There is also no change in potential energy. Then, the energy balance for a nozzle reduces as follows:

(87) $$-{\dot{Q}}_{\mathrm{loss}}=\dot{m}\left[\left(h_{2}-h_{1}\right)+\frac{V_{2}^{2}-V_{1}^{2}}{2}\right]\;\to \;V_{2}=\sqrt{2\left(h_{1}-h_{2}-{\dot{Q}}_{\mathrm{loss}}/\dot{m}\right)+V_{1}^{2}}$$

Therefore, the kinetic energy of the fluid in a nozzle increases as its enthalpy decreases.

The source of exergy supplied in a nozzle is the fluid stream. Taking the decrease in the exergy of the fluid flowing through the nozzle as the *exergy expended* results in $\eta_{\mathrm{ex}}={\dot{X}}_{Q_{\mathrm{loss}}}/({\dot{X}}_{1}-{\dot{X}}_{2})$ for exergy efficiency, which is meaningless. This equation resembles the exergy efficiency relation of a non-adiabatic valve.

A nozzle is installed in a system to increase the kinetic energy of the fluid at the expense of its enthalpy, and the exergy efficiency relation of a nozzle should reflect this objective as the thermal energy of the fluid stream is converted into kinetic energy. Therefore, it makes practical sense to take the decrease in the *thermal exergy* of the fluid as *exergy expended*, and the increase in the *kinetic exergy* together with exergy associated with heat loss, if any, as *exergy recovered*. Then the exergy efficiency of a nozzle can be expressed as follows:

(88) $$\eta_{\mathrm{ex}}=\frac{{\dot{X}}_{\mathrm{recovered}}}{{\dot{X}}_{\mathrm{expended}}}=\frac{\dot{∆KE}+{\dot{X}}_{Q\_\mathrm{loss}}}{{\left({\dot{X}}_{1}-{\dot{X}}_{2}\right)}_{\mathrm{thermal}}}=1-\frac{{\dot{X}}_{\mathrm{destroyed}}}{{\left({\dot{X}}_{1}-{\dot{X}}_{2}\right)}_{\mathrm{thermal}}}$$

where

(89) $${\dot{X}}_{\mathrm{expended}}={\left({\dot{X}}_{1}-{\dot{X}}_{2}\right)}_{\mathrm{thermal}}=\dot{m}[\left(h_{1}-h_{2}\right)-T_{0}\left(s_{1}-s_{2}\right)]$$

(90) $${\dot{X}}_{\mathrm{recovered}}=\dot{∆KE}+{\dot{X}}_{Q\_\mathrm{loss}}=\dot{m}(V_{2}^{2}-V_{1}^{2})/2+(1-T_{0}/T_{b}){\dot{Q}}_{\mathrm{loss}}$$

(91) $${\dot{X}}_{\mathrm{destroyed}}=T_{0}{\dot{S}}_{\mathrm{gen}}=T_{0}[\dot{m}(s_{2}-s_{1})+{\dot{Q}}_{\mathrm{loss}}/T_{b}].$$

For an adiabatic nozzle [${\dot{Q}}_{\mathrm{loss}}=0$ and thus ${\dot{S}}_{\mathrm{gen}}=\dot{m}(s_{2}-s_{1})$], it reduces to the following equation:

(92) $$\eta_{\mathrm{ex},\mathrm{adiabatic}}=\frac{\dot{∆KE}}{{\left({\dot{X}}_{1}-{\dot{X}}_{2}\right)}_{\mathrm{thermal}}}=\frac{(V_{2}^{2}-V_{1}^{2})/2}{\left(h_{1}-h_{2}\right)-T_{0}\left(s_{1}-s_{2}\right)}=\eta_{\mathrm{ex},\mathrm{extended}}$$

This is equivalent to the exergy efficiency of the *extended system* that includes the nozzle and its immediate surroundings since *T_b_* = *T*_0_ and ${\dot{X}}_{Q_{\mathrm{loss}}}$ = 0 for the extended system.

One might ask why the exergy associated with heat loss ${\dot{X}}_{Q\_\mathrm{loss}}$ is grouped with the increase in kinetic energy $\dot{∆KE}$ as exergy recovered since ${\dot{X}}_{Q\_\mathrm{loss}}$ is not an intended or desirable outcome and is eventually destroyed. One reason is bookkeeping: It is to satisfy the condition ${\dot{X}}_{\mathrm{destroyed}}={\dot{X}}_{\mathrm{expended}}-{\dot{X}}_{\mathrm{recovered}}$ and to ensure that the exergy efficiency of the system (the physical device) becomes 100% when there are no internal irreversibilities within the system and thus ${\dot{S}}_{\mathrm{gen}}=0$.

The other reason is that ${\dot{X}}_{Q\_\mathrm{loss}}$ is not necessarily a loss; it is a potential exergy source to produce work. For example, part or all of ${\dot{X}}_{Q\_\mathrm{loss}}$ can be converted into work by wrapping thermoelectric generators around the outer surfaces of a nozzle, thereby turning ${\dot{X}}_{Q\_\mathrm{loss}}$ into a valuable resource. When utilizing ${\dot{X}}_{Q\_\mathrm{loss}}$ is not feasible, it is allowed to vanish as heat loss to the environment; we can avoid this dilemma by analyzing the *extended system* instead of the *physical system* since ${\dot{X}}_{Q\_\mathrm{loss}}$ is destroyed in that case as in the immediate surroundings of the device and ${\dot{X}}_{Q\_\mathrm{loss}}$ becomes part of ${\dot{X}}_{\mathrm{destroyed}}$, leaving $\dot{∆KE}$ as the sole term as exergy recovered. The exergy efficiency in this case truly reflects *the percentage of the exergy supplied that is utilized as the intended outcome of the system*. A similar argument can be made for all devices involving heat loss to the environment.

As an alternative, we can take the exergy of the fluid at the *nozzle inlet* as the exergy expended, and take the exergy of the fluid *at the exit* together with ${\dot{X}}_{Q\_\mathrm{loss}}$ as the exergy recovered, and define exergy efficiency as their ratio as follows:

(93) $$\eta_{\mathrm{ex},\mathrm{alt}}=\frac{{\dot{X}}_{\mathrm{mass},\mathrm{out}}+{\dot{X}}_{Q\_\mathrm{loss}}}{{\dot{X}}_{\mathrm{mass},\mathrm{in}}}=\frac{{\dot{X}}_{2}+{\dot{X}}_{Q\_\mathrm{loss}}}{{\dot{X}}_{1}}=1-\frac{{\dot{X}}_{\mathrm{destroyed}}}{{\dot{X}}_{1}}$$

where

(94) $${\dot{X}}_{\mathrm{mass},\mathrm{in}}={\dot{X}}_{1}=\dot{m}[\left(h_{1}-h_{0}\right)-T_{0}\left(s_{1}-s_{0}\right)+V_{1}^{2}/2]$$

$${\dot{X}}_{\mathrm{mass},\mathrm{out}}={\dot{X}}_{2}=\dot{m}[\left(h_{2}-h_{0}\right)-T_{0}\left(s_{2}-s_{0}\right)+V_{2}^{2}/2]$$

(95) $${\dot{X}}_{\mathrm{destroyed}}=T_{0}{\dot{S}}_{\mathrm{gen}}=T_{0}[\dot{m}(s_{2}-s_{1})+{\dot{Q}}_{\mathrm{loss}}/T_{b}]$$

**Figure 6:**
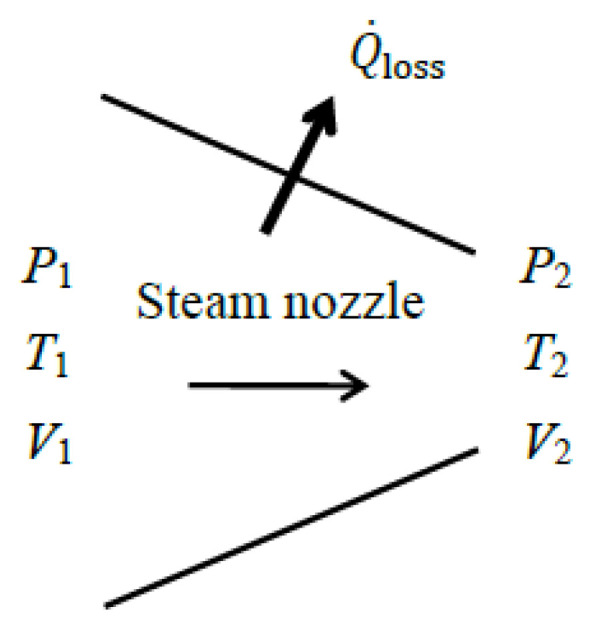
A nozzle with heat loss.

As a numerical application, we calculate the exergy efficiency of a steam nozzle with a mass flow rate of $\dot{m}=0.5\;\mathrm{kg}/s$, inlet conditions of *P*_1_ = 800 kPa, *T*_1_ = 400 °C and *V*_1_ = 10 m/s, exit conditions of *P*_2_ = 400 kPa and *T*_2_ = 350 °C, and a heat loss of ${\dot{Q}}_{\mathrm{loss}}=14\;\mathrm{kW}$ (Figure 6). We take the average temperature of the nozzle surface as *T_b_* = 350 °C.

We take the *physical nozzle* in steady operation as the system. The exit velocity of the steam is determined from the energy balance to be *V*_2_ = 373 m/s. Taking the decrease in the thermal exergy as *exergy expended* and the increase in the kinetic energy of steam together with exergy of heat loss as *exergy recovered*, we obtain ${\dot{X}}_{\mathrm{expended}}=508-434=73.6\;\mathrm{kW}$, ${\dot{X}}_{\mathrm{recovered}}=34.8-0.03+7.3=42.1\;\mathrm{kW}$, and *η*_ex_ = 42.1/73.6 = 0.57 or 57%. That is, 57% of the thermal exergy of the incoming steam is converted into kinetic energy at the nozzle exit plus exergy content of heat loss while the remaining 43% is destroyed within the nozzle. Using the value ${\dot{X}}_{\mathrm{destroyed}}=31.5\;\mathrm{kW}$ gives the same result for exergy efficiency, *η*_ex_ = 1 − 31.5/73.6 = 0.57. Note that of the 73.6 kW of exergy decrease associated with the enthalpy decrease in the fluid, 34.8 kW is converted to kinetic exergy, 7.3 kW is contained in the heat loss, and 31.5 kW is destroyed within the nozzle due to irreversibilities. For the extended system, the exergy of heat loss ${\dot{X}}_{\mathrm{loss}}=7.3\;\mathrm{kW}$ is destroyed within the immediate surroundings, increasing the exergy destruction from 31.5 kW to 38.8 kW. The exergy efficiency of the extended system is *η*_ex_ = 1 − 38.8/73.6 = 0.47.

Using the *alternative approach* of taking the total exergy of the fluid at the nozzle inlet as the *exergy expended* and the total exergy of the fluid at the nozzle exit plus the exergy of heat loss as the *exergy recovered*, we obtain ${\dot{X}}_{\mathrm{expended}}={\dot{X}}_{1}=508\;\mathrm{kW}$, ${\dot{X}}_{\mathrm{recovered}}={\dot{X}}_{2}+{\dot{X}}_{\mathrm{loss}}=469+7.3=476.3\;\mathrm{kW}$, and *η*_ex,alt_ = 476.3/508 = 0.94, which is considerably higher than the 57%. The exergy efficiency of the extended system in this case becomes *η*_ex,alt_ = 1 − 38.8/508 = 0.92.

The reason for the large difference in exergy efficiency between the two approaches is the magnitude of the exergy expended used in the denominator of the two exergy efficiency relations (73.6 vs. 508 kW): $\eta_{\mathrm{ex}}=1-{\dot{X}}_{\mathrm{destroyed}}/{\left({\dot{X}}_{1}-{\dot{X}}_{2}\right)}_{\mathrm{thermal}}=1-31.5/73.6=0.57$ and $\eta_{\mathrm{ex},\mathrm{alt}}=1-{\dot{X}}_{\mathrm{destroyed}}/{\dot{X}}_{1}=1-31.5/508=0.92$. Either approach can be used in practice to calculate exergy efficiency of a nozzle. We recommend using the first approach because it is more in line with the purpose of the device since the objective of a nozzle is to increase velocity by consuming enthalpy. Also, an *extended system* analysis should be preferred since the exergy of the heat loss from the nozzle is destined to be destroyed within the immediate surroundings as the temperature drops to the environment temperature. The numerator in this case contains only the kinetic energy term, which is more meaningful. The exergy efficiency of the extended system is lower (47% instead of 57%), but it reflects the reality better since the exergy of heat loss is destined to be destroyed. This attests that the nozzle recovers only 47% of the available exergy to increase the velocity of steam and destroys the remaining 53% via irreversibilities, which includes the irreversibility associated with heat loss (it constitutes 10 percentage points of 53%).

When the process is forced to be *reversible* by setting ${\dot{S}}_{\mathrm{gen}}=0$ and thus $s_{2}=s_{1}$ and lifting the restriction *T*_2_ = 350 °C at the nozzle exit (it is calculated to be *T*_2_ = 289 °C in the reversible case), both approaches give an exergy efficiency of *η*_ex_ =1, as expected, since ${\dot{X}}_{\mathrm{destroyed}}=T_{0}{\dot{S}}_{\mathrm{gen}}=0$ for both cases. If all irreversibilities were eliminated and the exergy efficiency were 100%, this nozzle would accelerate steam from 10 m/s to 624 m/s instead of 373 m/s, and the steam temperature at the nozzle exit would drop to 289 °C instead of 350 °C. This is thermodynamically best possible performance.

### 5.6. Diffusers

A diffuser’s objective is to slow down fluid and increase its pressure. Therefore, the arguments given above for nozzles are also valid for diffusers, as they perform opposite tasks. Like nozzles, a typical diffuser requires no work or heat transfer, except for heat loss to the environment ${\dot{Q}}_{\mathrm{loss}}$. There is also no change in potential energy. Thus, the energy balance for a diffuser reduces as follows:

(96) $$-{\dot{Q}}_{\mathrm{loss}}=\dot{m}\left[\left(h_{2}-h_{1}\right)+\frac{V_{2}^{2}-V_{1}^{2}}{2}\right]\;\to \;V_{2}=\sqrt{2\left(h_{1}-h_{2}-{\dot{Q}}_{\mathrm{loss}}/\dot{m}\right)+V_{1}^{2}}$$

Therefore, the enthalpy of the fluid in a diffuser increases as its kinetic energy decreases.

A diffuser is installed in a system to increase the enthalpy (and thus pressure) of the fluid at the expense of its kinetic energy, and the exergy efficiency relation of a diffuser should reflect this objective as the kinetic energy of the fluid is converted into enthalpy. Therefore, it makes practical sense to take the decrease in the fluid’s *kinetic energy* as *exergy expended*, and the increase in its *thermal exergy* together with exergy transfer associated with heat loss, if any, as *exergy recovered*. Then the exergy efficiency of a diffuser can be expressed as follows:

(97) $$\eta_{\mathrm{ex}}=\frac{{\dot{X}}_{\mathrm{recovered}}}{{\dot{X}}_{\mathrm{expended}}}=\frac{{\left({\dot{X}}_{2}-{\dot{X}}_{1}\right)}_{\mathrm{thermal}}+{\dot{X}}_{Q\_\mathrm{loss}}}{\dot{∆KE}}=1-\frac{{\dot{X}}_{\mathrm{destroyed}}}{\dot{∆KE}}$$

where

(98) $${\dot{X}}_{\mathrm{expended}}=\dot{∆KE}=\dot{m}(V_{1}^{2}-V_{2}^{2})/2$$

(99) $${\dot{X}}_{\mathrm{recovered}}={\left({\dot{X}}_{2}-{\dot{X}}_{1}\right)}_{\mathrm{thermal}}+{\dot{X}}_{Q\_\mathrm{loss}}\;\;\;\;\;\;\;\;\;\;\;\;\;\;\;\;\;\;=\dot{m}\left[\left(h_{2}-h_{1}\right)-T_{0}\left(s_{2}-s_{1}\right)\right]+(1-T_{0}/T_{b}){\dot{Q}}_{\mathrm{loss}}\;$$

(100) $${\dot{X}}_{\mathrm{destroyed}}=T_{0}{\dot{S}}_{\mathrm{gen}}=T_{0}[\dot{m}(s_{2}-s_{1})+{\dot{Q}}_{\mathrm{loss}}/T_{b}]$$

For an adiabatic diffuser [${\dot{Q}}_{\mathrm{loss}}=0$ and thus ${\dot{S}}_{\mathrm{gen}}=\dot{m}(s_{2}-s_{1})$] it reduces to the following equation:

(101) $$\eta_{\mathrm{ex},\mathrm{adiabatic}}=\frac{{\left({\dot{X}}_{2}-{\dot{X}}_{1}\right)}_{\mathrm{thermal}}}{\dot{∆KE}}=\frac{\left(h_{2}-h_{1}\right)-T_{0}\left(s_{2}-s_{1}\right)}{(V_{1}^{2}-V_{2}^{2})/2}=\eta_{\mathrm{ex},\mathrm{extended}}$$

This is equivalent to the exergy efficiency of the *extended system* that includes the diffuser and its immediate surroundings since *T_b_* = *T*_0_ and ${\dot{X}}_{Q_{\mathrm{loss}}}$ = 0 for an extended system.

As an alternative, we can take the exergy of the fluid at the *diffuser inlet* as the *exergy expended*, and take the exergy of the fluid *at the exit* together with ${\dot{X}}_{Q\_\mathrm{loss}}$ as *exergy recovered*, and define exergy efficiency as their ratio as follows:

(102) $$\eta_{\mathrm{ex},\mathrm{alt}}=\frac{{\dot{X}}_{\mathrm{mass},\mathrm{out}}+{\dot{X}}_{Q\_\mathrm{loss}}}{{\dot{X}}_{\mathrm{mass},\mathrm{in}}}=\frac{{\dot{X}}_{2}+{\dot{X}}_{Q\_\mathrm{loss}}}{{\dot{X}}_{1}}=1-\frac{{\dot{X}}_{\mathrm{destroyed}}}{{\dot{X}}_{1}}$$

where

(103) $${\dot{X}}_{\mathrm{mass},\mathrm{in}}={\dot{X}}_{1}=\dot{m}[\left(h_{1}-h_{0}\right)-T_{0}\left(s_{1}-s_{0}\right)+V_{1}^{2}/2]$$

(104) $${\dot{X}}_{\mathrm{mass},\mathrm{out}}={\dot{X}}_{2}=\dot{m}[\left(h_{2}-h_{0}\right)-T_{0}\left(s_{2}-s_{0}\right)+V_{2}^{2}/2]$$

(105) $${\dot{X}}_{\mathrm{destroyed}}=T_{0}{\dot{S}}_{\mathrm{gen}}=T_{0}[\dot{m}(s_{2}-s_{1})+{\dot{Q}}_{\mathrm{loss}}/T_{b}]$$

Note that these relations are identical to those obtained for a nozzle.

**Figure 7:**
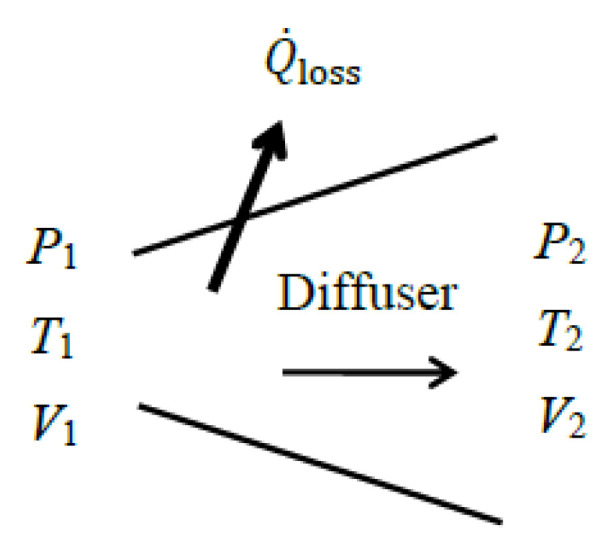
A diffuser with heat loss.

As a numerical application, we determine the exergy efficiency of a steam diffuser with a mass flow rate of $\dot{m}=0.5\;\mathrm{kg}/s$, inlet conditions of *P*_1_ = 150 kPa, *T*_1_ = 120 °C, and *V*_1_ = 550 m/s, and exit conditions of *P*_2_ = 300 kPa, *T*_2_ = 180 °C, and *V*_2_ = 160 m/s (Figure 7). Some heat is lost from the diffuser to its surroundings, and irreversibilities within the immediate surroundings are to be considered.

We take the diffuser in steady operation and its immediate surroundings as the *extended system*. Heat loss is determined from the energy balance to be ${\dot{Q}}_{\mathrm{loss}}=12.6\;kW$. Since *T_b_* = *T*_0_, exergy transfer associated with heat is zero for an extended system and thus ${\dot{X}}_{Q_{\mathrm{loss}}}$ = 0. Taking the decrease in the kinetic energy as *exergy expended* and the increase in the thermal exergy of steam as *exergy recovered*, we obtain ${\dot{X}}_{\mathrm{expended}}=\dot{m}(V_{1}^{2}-\;V_{2}^{2})/2\;=75.6-6.4=69.2\;\mathrm{kW}$, ${\dot{X}}_{\mathrm{recovered}}=338.2-274.7=63.5\;\mathrm{kW}$, and *η*_ex_ = 63.5/69.2 = 0.92 or 92%. That is, 92% of the kinetic exergy of the incoming steam is converted into thermal energy at the nozzle exit while the remaining 8% is destroyed within the nozzle and its immediate surroundings. Using the value ${\dot{X}}_{\mathrm{destroyed}}=5.7\;\mathrm{kW}$ gives the same result for exergy efficiency, *η*_ex_ = 1 − 5.7/69.2 = 0.92. Note that of the 69.2 kW of exergy decrease from the kinetic energy, 63.5 kW is converted to thermal exergy and 5.8 kW is destroyed within the nozzle and its immediate surroundings due to irreversibilities (of the 5.8 kW total exergy destruction, 2.0 kW occurs within the nozzle while the remaining 3.8 kW takes place within the immediate surroundings).

The *alternative approach* of taking the total exergy of the fluid at the diffuser inlet as the *exergy expended* and the total exergy of the fluid at the nozzle exit as the *exergy recovered*, we obtain ${\dot{X}}_{\mathrm{expended}}={\dot{X}}_{1}=350.3\;\mathrm{kW}$, ${\dot{X}}_{\mathrm{recovered}}={\dot{X}}_{2}+{\dot{X}}_{\mathrm{loss}}=344.6+0=344.6\;\mathrm{kW}$, and *η*_ex,alt_ = 344.6/350.3 = 0.98, which is somewhat higher than the 92%. Again, the reason for the difference between the two results (98% and 92%) is the magnitude of the base used in the denominator of the exergy efficiency relations, as discussed above for nozzles.

Both approaches are valid and either can be used in practice to calculate the exergy efficiency of a diffuser. However, the first approach should be preferred because it is more in line with the diffuser’s purpose, which is to increase enthalpy (and thus pressure) by consuming kinetic energy.

### 5.7. Heat Exchangers

**Figure 8:**
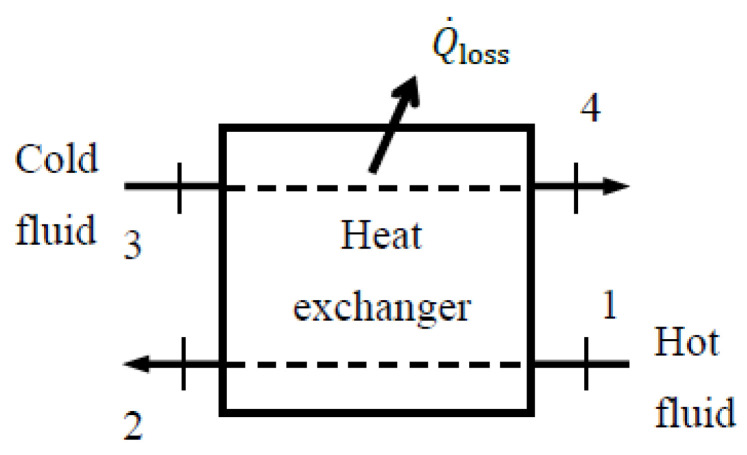
A heat exchanger.

Heat exchangers are typically well-insulated devices, so the changes in the kinetic and potential energy of the fluid streams are negligible. When determining the exergy efficiency of a heat exchanger with two unmixed fluid streams, the first thought that comes to mind is to take the hot fluid stream as the exergy resource (Figure 8), in which case the exergy of the cold stream increases at the expense of the hot stream. In this case, the exergy expended becomes the decrease in the exergy of the hot fluid, while the exergy recovered becomes the increase in the exergy of the cold fluid, plus ${\dot{X}}_{Q_{\mathrm{loss}}}$ when heat loss from the heat exchanger ${\dot{Q}}_{\mathrm{loss}}$ is not negligible. The ratio of the two gives exergy efficiency, and the difference between the two is exergy destroyed.

Denoting incoming hot and cold streams as 1 and 3, respectively, and the corresponding outgoing ones as 2 and 4, the exergy efficiency of a heat exchanger can be expressed as follows:

(106) $$\eta_{\mathrm{ex}}=\frac{{\dot{X}}_{\mathrm{recovered}}}{{\dot{X}}_{\mathrm{expended}}}=\frac{{\left({\dot{X}}_{4}-{\dot{X}}_{3}\right)}_{\mathrm{cold}}+{\dot{X}}_{Q\_\mathrm{loss}}}{{\left({\dot{X}}_{1}-{\dot{X}}_{2}\right)}_{\mathrm{hot}}}=1-\frac{{\dot{X}}_{\mathrm{destroyed}}}{{\left({\dot{X}}_{1}-{\dot{X}}_{2}\right)}_{\mathrm{hot}}}$$

where

(107) $${\dot{m}}_{1}={\dot{m}}_{2}={\dot{m}}_{\mathrm{hot}}\;\;\mathrm{and}\;\;{\dot{m}}_{3}={\dot{m}}_{4}={\dot{m}}_{\mathrm{cold}}\left(\mathrm{mass}\;\mathrm{balance}\right)$$

(108) $${\dot{m}}_{1}h_{1}+{\dot{m}}_{3}h_{3}={\dot{m}}_{2}h_{2}+{\dot{m}}_{4}h_{4}+{\dot{Q}}_{\mathrm{loss}}\left(\mathrm{energy}\;\mathrm{balance}\right)$$

(109) $${\dot{X}}_{i}={\dot{m}}_{i}[(h_{i}-h_{0})-T_{0}(s_{i}-s_{0})]\;\mathrm{for}\;i\;=\;1,\;2,\;3\;\mathrm{and}\;4\;\;(\mathrm{exergies}\;\mathrm{of}\;\mathrm{flow}\;\mathrm{streams})$$

(110) $${\dot{X}}_{Q\_\mathrm{loss}}={\dot{Q}}_{\mathrm{loss}}(1-T_{0}/T_{b})\;\;(\mathrm{exergy}\;\mathrm{transfer}\;\mathrm{by}\;\mathrm{heat}\;\mathrm{loss})$$

(111) $${\dot{X}}_{\mathrm{destroyed}}=T_{0}{\dot{S}}_{\mathrm{gen}}\;\mathrm{where}\;{\dot{S}}_{\mathrm{gen}}={\dot{m}}_{\mathrm{hot}}(s_{2}-s_{1})+{\dot{m}}_{\mathrm{cold}}(s_{4}-s_{3})+{\dot{Q}}_{\mathrm{loss}}/T_{b}$$

When heat loss from the heat exchanger is negligible (${\dot{X}}_{Q_{\mathrm{loss}}}=0$) for an *extended system* (${\dot{X}}_{Q_{\mathrm{loss}}}=0$) it can be expressed as follows:

(112) $$\eta_{\mathrm{ex},\mathrm{adiabatic}}=\frac{{\dot{X}}_{\mathrm{recovered}}}{{\dot{X}}_{\mathrm{expended}}}=\frac{{\left({\dot{X}}_{4}-{\dot{X}}_{3}\right)}_{\mathrm{cold}}}{{\left({\dot{X}}_{1}-{\dot{X}}_{2}\right)}_{\mathrm{hot}}}=1-\frac{{\dot{X}}_{\mathrm{destroyed}}}{{\left({\dot{X}}_{1}-{\dot{X}}_{2}\right)}_{\mathrm{hot}}}=\eta_{\mathrm{ex},\mathrm{expended}}$$

This approach seems reasonable but lacks generality: It is limited to heat exchangers, in which all heat transfer occurs at or above the environment temperature *T*_0_; that is, *T*_cold,in_ ≥ *T*_0_. For example, it is not applicable when the temperature of the cold stream remains below the environment temperature. In this case, the exergy of the cold stream will actually decrease with heat transfer instead of increasing, and there will be no exergy recovery.

A more general approach that is valid for all heat exchangers, including those used in the refrigeration systems, is to take the sum of the exergies of the two inlet streams as *exergy expended* and the sum of the exergies of the exit streams plus the exergy transfer associated with heat loss, if any, as *exergy recovered*. Exergy efficiency in this case can be expressed as follows:

(113) $$\eta_{\mathrm{ex}}=\frac{{\dot{X}}_{\mathrm{recovered}}}{{\dot{X}}_{\mathrm{expended}}}=\frac{{\dot{X}}_{2}+{\dot{X}}_{4}+{\dot{X}}_{Q\_\mathrm{loss}}}{{\dot{X}}_{1}+{\dot{X}}_{3}}=1-\frac{{\dot{X}}_{\mathrm{destroyed}}}{{\dot{X}}_{1}+{\dot{X}}_{3}}$$

For an adiabatic heat exchanger (${\dot{X}}_{Q_{\mathrm{loss}}}=0$) or an *extended system* (${\dot{X}}_{Q_{\mathrm{loss}}}=0$) it reduces as follows:

(114) $$\eta_{\mathrm{ex},\mathrm{adiabatic}}=\frac{{\dot{X}}_{\mathrm{recovered}}}{{\dot{X}}_{\mathrm{expended}}}=\frac{{\dot{X}}_{2}+{\dot{X}}_{4}}{{\dot{X}}_{1}+{\dot{X}}_{3}}=1-\frac{{\dot{X}}_{\mathrm{destroyed}}}{{\dot{X}}_{1}+{\dot{X}}_{3}}=\eta_{\mathrm{ex},\mathrm{extended}}$$

These relations can be used even when the cold stream experience exergy decreases, which can happen when the cold stream enters the heat exchanger at sub-environment temperatures. We recommend using this second approach when one or more streams are at sub-environmental temperatures. If all fluid streams are at or above the environment temperature, the first approach should be preferred since it is more intuitive.

When the heat exchanger is not adiabatic, it makes practical sense to analyze the *extended system*, which includes immediate surroundings of the device such that the boundaries of the extended system are at the environment temperature of *T*_0_ instead of the outer surface temperature *T_b_* of the device. This way, exergy associated with heat loss is completely destroyed and becomes zero as heat crosses the extended system boundary, ${\dot{X}}_{Q\_\mathrm{loss}}=0$. The exergy efficiency of the extended system reflects the effects of the irreversibilities that occur within and just outside the heat exchanger. This is a plausible approach since no attempt is made in practice to utilize the exergy associated with heat lost from the heat exchanger.

When there is heat loss from the heat exchanger, the exergy efficiency of the *extended system* will be somewhat lower than the exergy efficiency of the *device* taken as the system. This is because the former accounts for exergy destruction within the immediate surroundings of the device, while the latter does not. The extended system analysis provides realistic values for exergy destruction associated with the process. Thus, the exergy efficiency of the extended system can be considered the exergy efficiency of the *process*.

As a numerical application, we determine the exergy efficiency of an adiabatic water heat exchanger with ${\dot{m}}_{\mathrm{hot}}=0.5\;\mathrm{kg}/s$, *T*_1_ = 90 °C, ${\dot{m}}_{\mathrm{cold}}=0.8\;\mathrm{kg}/s$, *T*_3_ = 30 °C, *T*_4_ = 50 °C, and *P*_1_ = *P*_2_ = 200 kPa.

The heat exchanger is adiabatic and thus ${\dot{Q}}_{\mathrm{loss}}=0$ and ${\dot{X}}_{Q\_\mathrm{loss}}=0$. Then, the general exergy efficiency relation above gives ${\dot{X}}_{\mathrm{expended}}=13.2\;\mathrm{kW}$, ${\dot{X}}_{\mathrm{recovered}}=6.99\;\mathrm{kW}$, and *η*_ex_ = 0.528. That is, 52.8% of the exergy content of the incoming hot and cold water streams are retained in the outgoing hot and cold water streams in this case. The remaining 47.2% of incoming exergy is destroyed within the heat exchanger during this heat exchange process. Using ${\dot{X}}_{\mathrm{destroyed}}=6.24\;\mathrm{kW}$ in the calculations of exergy efficiency gives the same result.

If the heat exchanger were not adiabatic and there was a heat loss of ${\dot{Q}}_{\mathrm{loss}}=3.8$ kW we would obtain, for the *extended system*, ${\dot{X}}_{\mathrm{expended}}=13.2\;\mathrm{kW}$, ${\dot{X}}_{\mathrm{recovered}}=6.62\;\mathrm{kW}$, and *η*_ex,extended_ = 0.50. Exergy destruction in this case would be ${\dot{X}}_{\mathrm{destroyed}}=6.62\;\mathrm{kW}$.

### 5.8. Mixing Chambers

**Figure 9:**
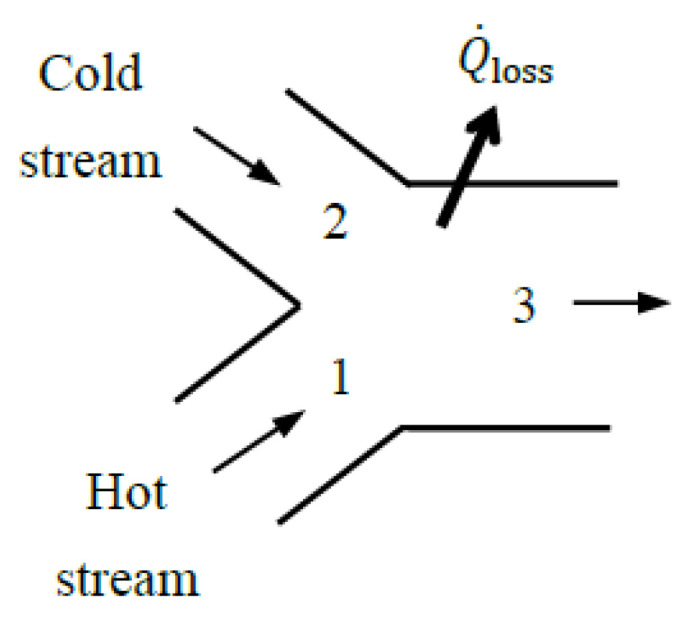
A mixing chamber that involves heat loss.

A mixing chamber is a special type of heat exchanger in which hot and cold fluids mix and exit the device as a single stream, as shown in Figure 9. Therefore, the arguments given above for heat exchangers are also valid for mixing chambers. Using the general approach for heat exchangers and thus taking the sum of the exergies of the two inlet streams as *exergy expended* and the exergy of the exit stream plus the exergy transfer associated with heat loss, if any, as *exergy recovered*, the exergy efficiency of a mixing chamber is expressed as follows:

(115) $$\eta_{\mathrm{ex}}=\frac{{\dot{X}}_{\mathrm{recovered}}}{{\dot{X}}_{\mathrm{expended}}}=\frac{{\dot{X}}_{3}+{\dot{X}}_{Q\_\mathrm{loss}}}{{\dot{X}}_{1}+{\dot{X}}_{2}}=1-\frac{{\dot{X}}_{\mathrm{destroyed}}}{{\dot{X}}_{1}+{\dot{X}}_{2}}$$

where

(116) $${\dot{m}}_{1}+{\dot{m}}_{2}={\dot{m}}_{3}\;\;\;(\mathrm{mass}\;\mathrm{balance})$$

(117) $${\dot{m}}_{1}h_{1}+{\dot{m}}_{2}h_{2}={\dot{m}}_{3}h_{3}+{\dot{Q}}_{\mathrm{loss}}\;\;\;(\mathrm{energy}\;\mathrm{balance})$$

(118) $${\dot{X}}_{i}={\dot{m}}_{i}[(h_{i}-h_{0})-T_{0}(s_{i}-s_{0})]\;\mathrm{for}\;i\;=\;1,\;2\;\mathrm{and}\;3\;(\mathrm{exergies}\;\mathrm{of}\;\mathrm{flow}\;\mathrm{streams})$$

(119) $${\dot{X}}_{Q\_\mathrm{loss}}={\dot{Q}}_{\mathrm{loss}}(1-T_{0}/T_{b})\;(\mathrm{exergy}\;\mathrm{transfer}\;\mathrm{that}\;\mathrm{accompany}\;\mathrm{heat}\;\mathrm{loss})$$

(120) $${\dot{X}}_{\mathrm{destroyed}}=T_{0}{\dot{S}}_{\mathrm{gen}}\;\mathrm{and}\;{\dot{S}}_{\mathrm{gen}}={\dot{m}}_{3}s_{3}-{\dot{m}}_{1}s_{1}-{\dot{m}}_{2}s_{2}+{\dot{Q}}_{\mathrm{loss}}/T_{b}$$

As a numerical application, we determine the exergy efficiency of a water mixing chamber with ${\dot{m}}_{\mathrm{hot}}=0.5\;\mathrm{kg}/s$, *T*_1_ = 90 °C, ${\dot{m}}_{\mathrm{cold}}=0.8\;\mathrm{kg}/s$, *T*_2_ = 30 °C, *P*_1_ = *P*_2_ = 200 kPa, and ${\dot{Q}}_{\mathrm{loss}}=0.65\;\mathrm{kW}$.

The mass and energy balance relations give *T*_3_ = 53 °C for the mixture temperature. Taking the mixing chamber and its immediate surroundings as the *extended system* so that the boundary temperature is the environment temperature *T*_0_ and thus ${\dot{X}}_{Q\_\mathrm{loss}}=0$, the relations above give ${\dot{X}}_{\mathrm{expended}}=13.23\;\mathrm{kW}$, ${\dot{X}}_{\mathrm{recovered}}=6.82\;\mathrm{kW}$, and *η*_ex,extended_ = 0.515. Using ${\dot{X}}_{\mathrm{destroyed}}=6.42\;\mathrm{kW}$ in calculations gives the same result for exergy efficiency. That is, 51.5% of the exergy content of the incoming hot and cold water streams are retained in the outgoing mixture in this case. The remaining 48.5% of incoming exergy is destroyed within the mixing chamber and its immediate surroundings. The exergy efficiency of the process is identical to the exergy efficiency of the extended system.

We now take the *mixing chamber* as the system. Taking the boundary temperature to be the mixture temperature, the relations above give ${\dot{X}}_{\mathrm{expended}}=13.23\;\mathrm{kW}$, ${\dot{X}}_{\mathrm{recovered}}=6.87\;\mathrm{kW}$, and *η*_ex_ = 0.519. The total entropy generation associated with this process is $\dot{S}$_gen_= 0.0215 kW/K, of which 0.0214 kW/K occurs within the mixing chamber and the remaining 0.0001 kW/K occurs within its immediate surroundings. Therefore, considering exergy destruction within the immediate surroundings of the mixing chamber reduces exergy efficiency from 51.9% to 51.5%. If no effort is made to utilize the heat at 53 °C lost to the surroundings, the exergy efficiency of 51.5% for the extended system is a more realistic measure of performance than the 51.9% for the mixing chamber alone since the entire exergy content of the lost heat will inevitably be destroyed.

### 5.9. Electric Motors and Generators

Electric motors convert electrical energy into mechanical energy, and generators convert mechanical energy into electrical energy. However, both mechanical and electrical energies are forms of work, and thus their exergy content is the same as their energy content. Therefore, for an extended system that includes the motor or generator and its immediate surroundings, the exergy efficiency of a motor or generator becomes equal to its energy efficiency.

Electric *generators* involve the conversion of mechanical energy to electrical energy, and their energy and exergy efficiencies are defined as the ratio of the electrical energy output to the mechanical energy input:

(121) $$\eta_{\mathrm{ex},\mathrm{generator}}=\frac{\mathrm{Exergy}\;\mathrm{recovered}}{\mathrm{Exergy}\;\mathrm{expended}}=\frac{\mathrm{Electrical}\;\mathrm{energy}\;\mathrm{output}}{\mathrm{Mechanical}\;\mathrm{energy}\;\mathrm{input}}=\frac{W_{\mathrm{elect},\mathrm{out}}}{W_{\mathrm{mech},\mathrm{in}}}\;=\eta_{\mathrm{energy},\mathrm{generator}}\;\;\;\;\;\;\;\;\;$$

Electric *motors* involve the conversion of electrical energy to mechanical energy, and when no attempt is made to utilize the heat lost, their energy and exergy efficiencies are defined as the ratio of the mechanical energy output to the electrical energy input:

(122) $$\eta_{\mathrm{ex},\mathrm{motor}}=\frac{\mathrm{Exergy}\;\mathrm{recovered}}{\mathrm{Exergy}\;\mathrm{expended}}=\frac{\mathrm{Mechanical}\;\mathrm{energy}\;\mathrm{output}}{\mathrm{Electrical}\;\mathrm{energy}\;\mathrm{input}}=\frac{W_{\mathrm{mech},\mathrm{out}}}{W_{\mathrm{elect},\mathrm{in}}}\;=\eta_{\mathrm{energy},\mathrm{motor}}\;\;\;\;\;\;\;\;\;$$

## 6. Exergy Efficiency of Heat Engines

**Figure 10:**
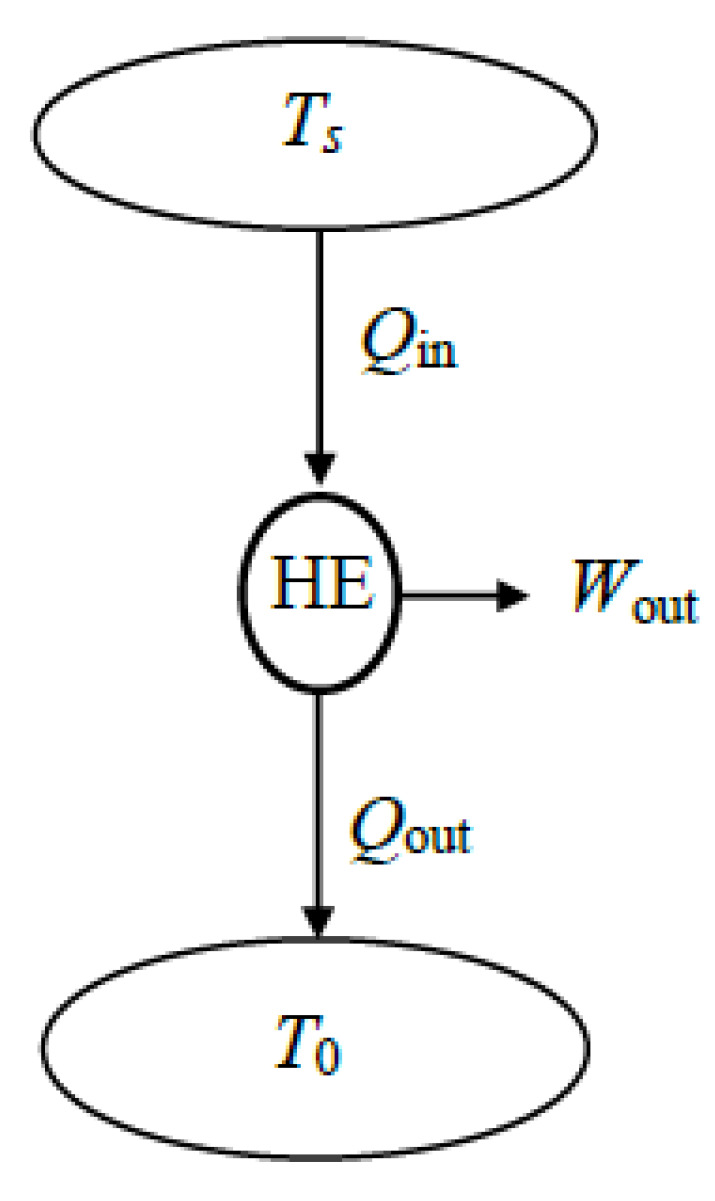
A heat engine operating between a source and the environment.

A major application area of exergy or second-law analysis is power plants, where electric power is generated by supplying high-temperature heat to the plant and rejecting low-temperature waste heat to the environment (Figure 10). The primary source of heat can be coal, oil, natural gas, biofuel, nuclear fuel, solar thermal energy, or geothermal energy, while the sink for waste heat can be atmospheric air, a river, a lake, the sea, or underground water.

The exergy efficiency of a power plant depends on whether the energy conversion processes such as combustion and generators are included as part of the system. Heat input can be the heating value of the fuel or the heat transferred to the working fluid, depending on how the system is selected. Also, the net work output can be mechanical or electrical net work output. When the extended system is used in exergy analysis, exergy loss is associated with heat loss from the system and its components become zero since heat leaves the extended system at the environment temperature and thus with zero exergy. This simplifies the analysis.

For a typical power plant, the exergy of heat supplied to the power plant constitutes the expended exergy and the actual net work output constitutes the recovered exergy. Then the exergy efficiency of a power plant can be expressed as follows:

(123) $${\dot{X}}_{\mathrm{expended}}={\dot{X}}_{\mathrm{input}}={\dot{X}}_{Q\_\mathrm{in}}={\dot{Q}}_{\mathrm{in}}\left(1-T_{0}/T_{s}\right)$$

(124) $${\dot{X}}_{\mathrm{recovered}}={\dot{X}}_{\mathrm{output}}={\dot{X}}_{W\_\mathrm{out}}={\dot{W}}_{\mathrm{act},\mathrm{out}}$$

(125) $$\eta_{\mathrm{ex}}=\frac{{\dot{X}}_{\mathrm{recovered}}}{{\dot{X}}_{\mathrm{expended}}}=\frac{{\dot{X}}_{\mathrm{output}}}{{\dot{X}}_{\mathrm{input}}}=\frac{{\dot{W}}_{\mathrm{act},\mathrm{out}}}{{\dot{Q}}_{\mathrm{in}}\left(1-T_{0}/T_{s}\right)}$$

or

(126) $$\eta_{\mathrm{ex}}=1-\frac{{\dot{X}}_{\mathrm{destroyed}}}{{\dot{X}}_{\mathrm{expended}}}=1-\frac{T_{0}{\dot{S}}_{\mathrm{gen}}}{{\dot{Q}}_{\mathrm{in}}\left(1-T_{0}/T_{s}\right)}$$

Exergy efficiency can be expressed in terms of thermal efficiency as follows:

(127) $$\eta_{\mathrm{ex}}=\frac{{\dot{W}}_{\mathrm{act},\mathrm{out}}}{{\dot{Q}}_{\mathrm{in}}\left(1-T_{0}/T_{s}\right)}=\frac{{\dot{W}}_{\mathrm{act},\mathrm{out}}}{{\dot{W}}_{\mathrm{rev},\mathrm{out}}}=\frac{{\dot{W}}_{\mathrm{act},\mathrm{out}}/{\dot{Q}}_{\mathrm{in}}}{{\dot{W}}_{\mathrm{rev},\mathrm{out}}/{\dot{Q}}_{\mathrm{in}}}=\frac{\eta_{\mathrm{th},\mathrm{act}}}{\eta_{\mathrm{th},\mathrm{rev}}}$$

since ${\dot{W}}_{\mathrm{rev},\mathrm{out}}={\dot{Q}}_{\mathrm{in}}(1-T_{0}/T_{s})$, $\eta_{\mathrm{th},\mathrm{act}}={\dot{W}}_{\mathrm{act},\mathrm{out}}/{\dot{Q}}_{\mathrm{in}}$, $\eta_{\mathrm{th},\mathrm{rev}}={\dot{W}}_{\mathrm{rev},\mathrm{out}}/{\dot{Q}}_{\mathrm{in}}$, and $\eta_{\mathrm{th},\mathrm{rev}}=1-T_{0}/T_{s}$, which is the thermal efficiency of a reversible heat engine operating between the temperature limits of T_s_ and T_0_. Therefore, the ratio of the actual thermal efficiency of a power plant to the reversible thermal efficiency, or the ratio of the actual work output of a power plant to the reversible work output gives the exergy efficiency of a power plant. To summarize, we have five exergy efficiency relations for power cycles, and all are equivalent to each other:

(128) $$\eta_{\mathrm{ex}}=\frac{{\dot{X}}_{\mathrm{recovered}}}{{\dot{X}}_{\mathrm{expended}}}=\frac{{\dot{X}}_{\mathrm{output}}}{{\dot{X}}_{\mathrm{input}}}=1-\frac{{\dot{X}}_{\mathrm{destroyed}}}{{\dot{X}}_{\mathrm{expended}}}=\frac{{\dot{W}}_{\mathrm{act},\mathrm{out}}}{{\dot{W}}_{\mathrm{rev},\mathrm{out}}}=\frac{\eta_{\mathrm{th},\mathrm{act}}}{\eta_{\mathrm{th},\mathrm{rev}}}$$

**Figure 11:**
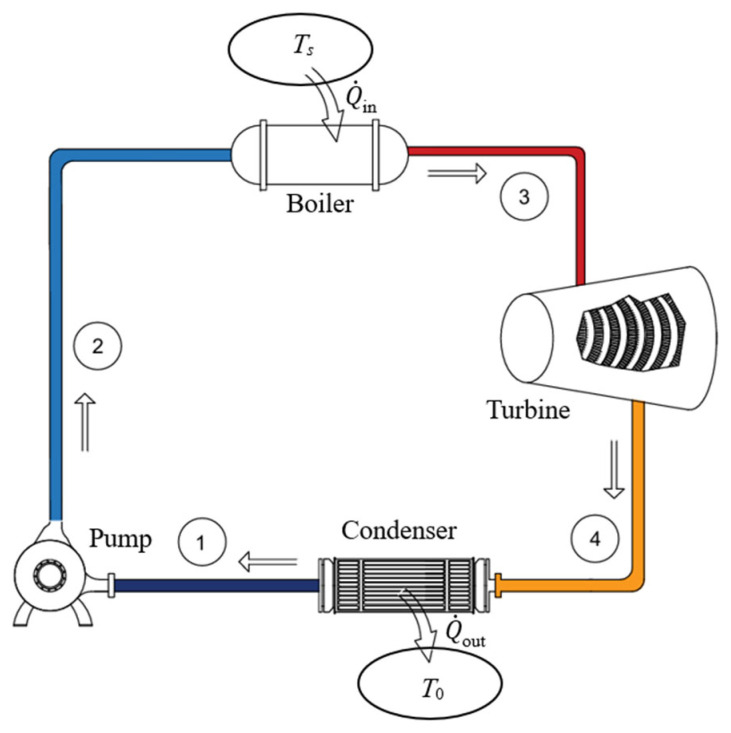
A simple steam power plant.

As a numerical application, we determine the exergy efficiency of a power plant operating on the simple Rankine cycle with steam mass flow rate of $\dot{m}=40\;\mathrm{kg}/s$. Steam leaves the boiler and enters the turbine at 5 MPa and 600 °C. The furnace temperature is *T_s_* = 1000 °C and the condenser pressure is 10 kPa (Figure 11). The turbine and pump are isentropic.

We take the steam power plant in steady operation and its immediate surroundings as the extended system. Using the relations above, we obtain ${\dot{Q}}_{\mathrm{in}}=139\;\mathrm{MW}$, ${\dot{Q}}_{\mathrm{out}}=84.4\;\mathrm{MW}$, *η*_th,act_ = 0.392, ${\dot{X}}_{\mathrm{recovered}}={\dot{W}}_{\mathrm{act},\mathrm{out}}=54.5\;\mathrm{MW}$, ${\dot{X}}_{\mathrm{expended}}=106.3\;\mathrm{MW}$, and *η*_ex_ $={\dot{X}}_{\mathrm{recovered}}/{\dot{X}}_{\mathrm{expended}}=0.512$. Using the value of exergy destroyed ${\dot{X}}_{\mathrm{destroyed}}=51.8\;\mathrm{MW}$ gives the same result for exergy efficiency. The reversible thermal efficiency for this plant is *η*_th,rev_ = 0.766 which gives *η*_ex_ = *η*_th,rev/_*η*_th,rev_ = 0.392/0.766 = 0.512, as expected. Therefore, 51.2% of the exergy consumed by the plant is recovered as shaft work while the remaining 48.8% is destroyed.

## 7. Exergy Efficiency of Refrigerators and Heat Pumps

**Figure 12:**
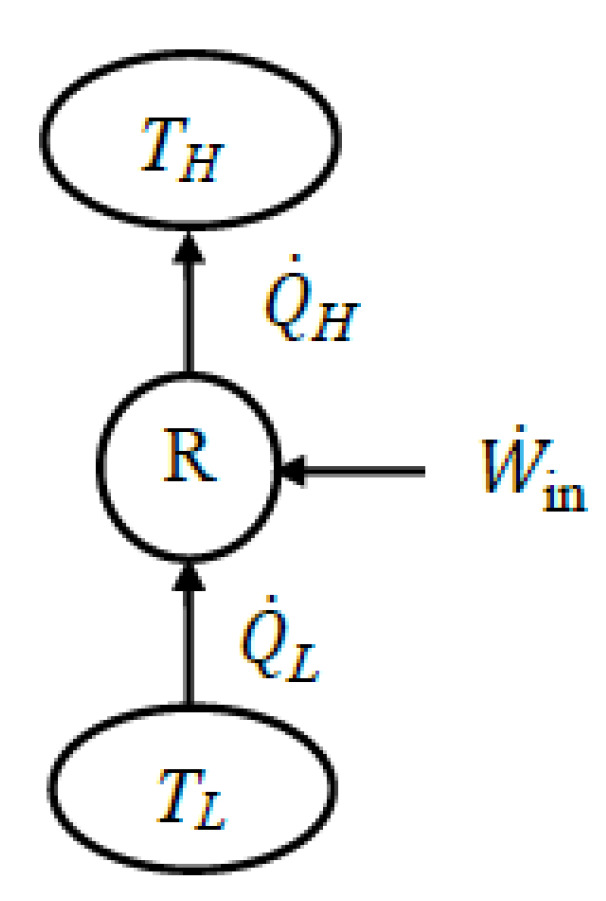
A refrigerator or heat pump.

Another major application area of exergy analysis is refrigerators and heat pumps where electric power is used to absorb heat from a low-temperature medium at *T_L_* and to reject heat to a high-temperature one at *T_H_* (Figure 12).

### 7.1. Refrigerators

Refrigerators (and air-conditioners) usually reject heat to the environment at *T_H_* = *T*_0_. When an extended system is analyzed, the rejected heat leaves the system at the environment temperature and thus with zero exergy. For a typical refrigerator, the power input constitutes the expended exergy and the exergy of the heat absorbed from the low-temperature medium constitutes the recovered exergy. Then, the exergy efficiency of a refrigerator can be expressed as follows:

(129) $${\dot{X}}_{\mathrm{expended}}={\dot{X}}_{\mathrm{input}}={\dot{X}}_{W\_\mathrm{in}}={\dot{W}}_{\mathrm{act},\mathrm{in}}$$

(130) $${\dot{X}}_{\mathrm{recovered}}={\dot{X}}_{\mathrm{output}}={\dot{X}}_{Q\_L}=-{\dot{Q}}_{L}\left(1-\frac{T_{0}}{T_{L}}\right)={\dot{Q}}_{L}\left(\frac{T_{0}-T_{L}}{T_{L}}\right)$$

(131) $$\eta_{\mathrm{ex}}=\frac{{\dot{X}}_{\mathrm{recovered}}}{{\dot{X}}_{\mathrm{expended}}}=\frac{{\dot{X}}_{\mathrm{output}}}{{\dot{X}}_{\mathrm{input}}}=\frac{{\dot{Q}}_{L}}{{\dot{W}}_{\mathrm{act},\mathrm{in}}}\left(\frac{T_{0}-T_{L}}{T_{L}}\right)$$

or

(132) $$\eta_{\mathrm{ex}}=1-\frac{{\dot{X}}_{\mathrm{destroyed}}}{{\dot{X}}_{\mathrm{expended}}}=1-\frac{T_{0}{\dot{S}}_{\mathrm{gen}}}{{\dot{W}}_{\mathrm{act},\mathrm{in}}}$$

where *T_L_* is the temperature of the refrigerated space, *T*_0_ is the environment temperature, and ${\dot{S}}_{\mathrm{gen}}={\dot{Q}}_{H}/T_{0}-{\dot{Q}}_{L}/T_{L}$ is entropy generation. Here the energy balance gives ${\dot{Q}}_{H}={\dot{Q}}_{L}+{\dot{W}}_{\mathrm{act},\mathrm{in}}$. Exergy efficiency can be expressed in terms of COP as follows:

(133) $$\eta_{\mathrm{ex}}=\frac{{\dot{Q}}_{L}}{{\dot{W}}_{\mathrm{act},\mathrm{in}}}\left(\frac{T_{0}-T_{L}}{T_{L}}\right)=\frac{{\dot{W}}_{\mathrm{rev},\mathrm{in}}}{{\dot{W}}_{\mathrm{act},\mathrm{in}}}=\frac{{\dot{Q}}_{L}/{COP}_{R,\mathrm{rev}}}{{\dot{Q}}_{L}/{COP}_{R,\mathrm{act}}}=\frac{{COP}_{R,\mathrm{act}}}{{COP}_{R,\mathrm{rev}}}$$

since ${\dot{W}}_{\mathrm{rev},\mathrm{in}}={\dot{Q}}_{L}/{COP}_{R,\mathrm{rev}}={\dot{Q}}_{L}(T_{0}-T_{L})/T_{L}$, ${COP}_{R,\mathrm{act}}={\dot{Q}}_{L}/{\dot{W}}_{act,in}$, and ${COP}_{R,\mathrm{rev}}=T_{L}/(T_{0}-T_{L})$, which is the COP of a reversible refrigerator operating between the temperature limits of *T_L_* and *T*_0_. Therefore, the ratio of the actual COP of a refrigerator to the COP of a reversible refrigerator or the ratio of the reversible work input to the actual work input gives the exergy efficiency of a refrigerator. To summarize, we have five exergy efficiency relations for refrigerators, and all are equivalent to each other:

(134) $$\eta_{\mathrm{ex}}=\frac{{\dot{X}}_{\mathrm{recovered}}}{{\dot{X}}_{\mathrm{expended}}}=\frac{{\dot{X}}_{\mathrm{output}}}{{\dot{X}}_{\mathrm{input}}}=1-\frac{{\dot{X}}_{\mathrm{destroyed}}}{{\dot{X}}_{\mathrm{expended}}}=\frac{{\dot{W}}_{\mathrm{rev},\mathrm{in}}}{{\dot{W}}_{\mathrm{act},\mathrm{in}}}=\frac{{COP}_{R,\mathrm{act}}}{{COP}_{R,\mathrm{rev}}}$$

**Figure 13:**
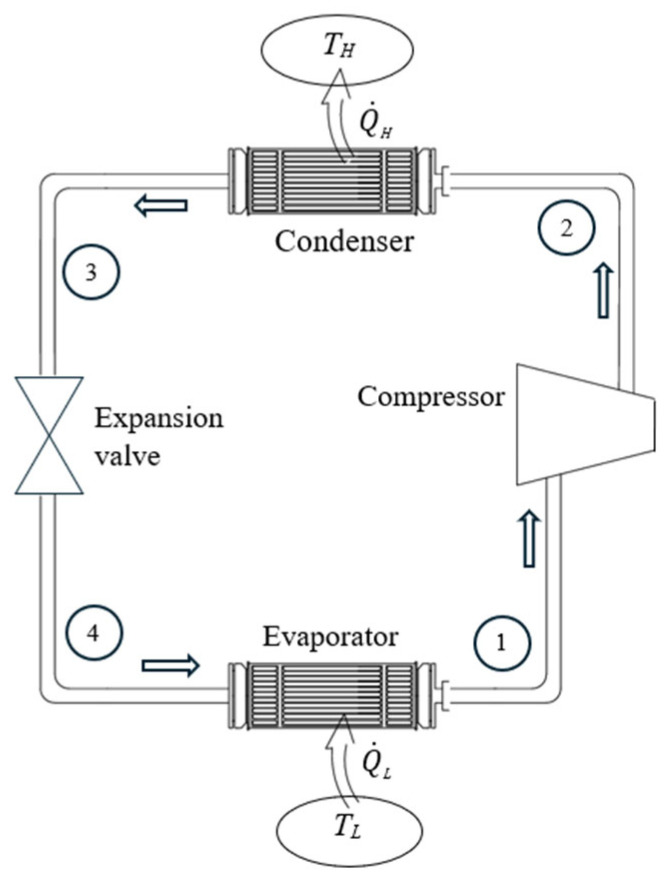
The simple vapor-compression refrigeration cycle.

As a numerical application, we determine the exergy efficiency of a refrigerator operating on the simple vapor-compression cycle with refrigerant-134a as the working fluid (Figure 13). The condenser and evaporator pressures are 800 kPa and 120 kPa, respectively, the compressor exit temperature is 50 °C, and the refrigerant flow rate is 0.5 kg/s. The compressor is isentropic. The temperature of the low-temperature medium is *T_L_* = –10 °C and the environment temperature is *T_H_* = *T*_0_ = 25 °C.

We take the refrigerator in steady operation and its immediate surroundings as the *extended system*. Using the relations above, we obtain ${\dot{Q}}_{L}=70.8\;\mathrm{kW}$, ${\dot{Q}}_{H}=90.5\;\mathrm{kW}$, COP_R,act_ = 3.59, ${\dot{X}}_{\mathrm{expended}}=19.7\;\mathrm{kW}$, ${\dot{X}}_{\mathrm{recovered}}={\dot{X}}_{Q\_L}=9.42\;\mathrm{kW}$, and *η*_ex_ = 0.478. Using the value of exergy destroyed gives the same result for exergy efficiency: ${\dot{S}}_{\mathrm{gen}}={\dot{Q}}_{H}/T_{0}-{\dot{Q}}_{L}/T_{L}=0.0345\;\mathrm{kW}/K$, ${\dot{X}}_{\mathrm{destroyed}}=T_{0}{\dot{S}}_{\mathrm{gen}}=10.3\;\mathrm{kW}$, and $\eta_{\mathrm{ex}}=1-{\dot{X}}_{\mathrm{destroyed}}/{\dot{X}}_{\mathrm{expended}}=0.478$. The COP of a reversible refrigerator operating between the same temperature limits is COP_R,rev_ = 7.51, which gives *η*_ex_ = COP_R,act_/COP_R,rev_ = 3.59/7.51= 0.478, as expected. Therefore, 47.8% of the exergy consumed by the refrigerator is recovered and stored in the cooled medium while the remaining 52.2% is destroyed.

### 7.2. Heat Pumps

We can repeat the above analysis for heat pumps whose primary objective is to heat a warm medium at temperature *T_H_* by absorbing heat from the cold environment at *T_L_* = *T*_0_. When an extended system is analyzed, heat is absorbed from the environment at temperature *T*_0_ and thus *Q_L_* is accommodated by zero exergy.

For a typical heat pump, the power input constitutes the expended exergy, and the exergy of the heat supplied to the high-temperature medium constitutes the recovered exergy. Then the exergy efficiency of a heat pump can be expressed as follows:

(135) $${\dot{X}}_{\mathrm{expended}}={\dot{X}}_{\mathrm{input}}={\dot{X}}_{W\_\mathrm{in}}={\dot{W}}_{\mathrm{act},\mathrm{in}}$$

(136) $${\dot{X}}_{\mathrm{recovered}}={\dot{X}}_{\mathrm{output}}={\dot{X}}_{Q\_H}={\dot{Q}}_{H}\left(1-\frac{T_{0}}{T_{H}}\right)={\dot{Q}}_{H}\left(\frac{T_{H}-T_{0}}{T_{H}}\right)$$

(137) $$\eta_{\mathrm{ex}}=\frac{{\dot{X}}_{\mathrm{recovered}}}{{\dot{X}}_{\mathrm{expended}}}=\frac{{\dot{X}}_{\mathrm{output}}}{{\dot{X}}_{\mathrm{input}}}=\frac{{\dot{Q}}_{H}}{{\dot{W}}_{\mathrm{act},\mathrm{in}}}\left(1-\frac{T_{0}}{T_{H}}\right)$$

or

(138) $$\eta_{\mathrm{ex}}=1-\frac{{\dot{X}}_{\mathrm{destroyed}}}{{\dot{X}}_{\mathrm{expended}}}=1-\frac{T_{0}{\dot{S}}_{\mathrm{gen}}}{{\dot{W}}_{\mathrm{act},\mathrm{in}}}$$

where *T_H_* is the temperature of the heated medium and ${\dot{S}}_{\mathrm{gen}}={\dot{Q}}_{H}/T_{H}-{\dot{Q}}_{L}/T_{0}$ is entropy generation. Here ${\dot{Q}}_{H}={\dot{Q}}_{L}+{\dot{W}}_{\mathrm{act},\mathrm{in}}$. Exergy efficiency can be expressed in terms of COP as follows:

(139) $$\eta_{\mathrm{ex}}=\frac{{\dot{Q}}_{H}}{{\dot{W}}_{\mathrm{act},\mathrm{in}}}\left(\frac{T_{H}-T_{0}}{T_{H}}\right)=\frac{{\dot{W}}_{\mathrm{rev},\mathrm{in}}}{{\dot{W}}_{\mathrm{act},\mathrm{in}}}=\frac{{\dot{Q}}_{H}/{COP}_{\mathrm{HP},\mathrm{rev}}}{{\dot{Q}}_{H}/{COP}_{\mathrm{HP},\mathrm{act}}}=\frac{{COP}_{\mathrm{HP},\mathrm{act}}}{{COP}_{\mathrm{HP},\mathrm{rev}}}$$

since ${\dot{W}}_{\mathrm{rev},\mathrm{in}}={\dot{Q}}_{H}/{COP}_{\mathrm{HP},\mathrm{rev}}={\dot{Q}}_{H}[(T_{H}-T_{0})/T_{H}]$, ${COP}_{\mathrm{HP},\mathrm{act}}={\dot{Q}}_{H}/{\dot{W}}_{act,in}$ and ${COP}_{\mathrm{HP},\mathrm{rev}}=T_{H}/(T_{H}-T_{0}),$ which is the COP of a reversible heat pump operating between the temperature limits of *T_H_* and *T*_0_. Therefore, the ratio of the actual COP of a heat pump to the COP of a reversible heat pump or the ratio of the reversible work input to the actual work input gives the exergy efficiency of a heat pump. To summarize, we have five exergy efficiency relations for heat pumps, and all are equivalent to each other:

(140) $$\eta_{\mathrm{ex}}=\frac{{\dot{X}}_{\mathrm{recovered}}}{{\dot{X}}_{\mathrm{expended}}}=\frac{{\dot{X}}_{\mathrm{output}}}{{\dot{X}}_{\mathrm{input}}}=1-\frac{{\dot{X}}_{\mathrm{destroyed}}}{{\dot{X}}_{\mathrm{expended}}}=\frac{{\dot{W}}_{\mathrm{rev},\mathrm{in}}}{{\dot{W}}_{\mathrm{act},\mathrm{in}}}=\frac{{COP}_{\mathrm{HP},\mathrm{act}}}{{COP}_{\mathrm{HP},\mathrm{rev}}}$$

As a numerical application, we determine the exergy efficiency of a heat pump operating on the simple ideal vapor-compression cycle with refrigerant-134a as the working fluid. The condenser and evaporator pressures are 800 kPa and 120 kPa, respectively, the compressor exit temperature is 50 °C, and the refrigerant flow rate is 0.5 kg/s. The temperature of the heated high-temperature medium is *T_H_* = 26 °C and the temperature of the cold environment that serves as the heat source is *T_L_* = *T*_0_ = 13 °C.

We take the heat pump in steady operation and its immediate surroundings as the *extended system*. Using the relations above, we obtain ${\dot{Q}}_{L}=70.8\;\mathrm{kW}$, ${\dot{Q}}_{H}=90.5\;\mathrm{kW}$, COP_HP_ = 4.59, ${\dot{X}}_{\mathrm{expended}}={\dot{W}}_{\mathrm{act},\mathrm{in}}=19.7\;\mathrm{kW}$, ${\dot{X}}_{\mathrm{recovered}}={\dot{X}}_{Q\_H}=3.93\;\mathrm{kW}$, COP_HP,rev_ = 23.0, and *η*_ex_ = 0.200. Using the value of exergy destroyed gives the same result for exergy efficiency: ${\dot{S}}_{\mathrm{gen}}={\dot{Q}}_{H}/T_{H}-{\dot{Q}}_{L}/T_{0}=0.551\;\mathrm{kW}/K$, ${\dot{X}}_{\mathrm{destroyed}}=T_{0}{\dot{S}}_{\mathrm{gen}}=15.8\;\mathrm{kW}$, and $\eta_{\mathrm{ex}}=1-{\dot{X}}_{\mathrm{destroyed}}/{\dot{X}}_{\mathrm{expended}}=0.200$. The COP of a reversible heat pump operating between the same temperature limits is COP_HP,rev_ = 23.0, which gives *η*_ex_ = COP_HP,act_/COP_HP,rev_ = 4.59/23= 0.200, as expected.

## 8. Conclusions

In this paper, exergy efficiency relations are developed for steady-flow devices such as turbines, compressors, nozzles, diffusers, valves, pipes and ducts, heat exchangers, mixing chambers, and cyclic devices such as heat engines, refrigerators, and heat pumps.

In engineering practice, the best measure of performance of energy systems is the exergy efficiency associated with the *process*, which includes the internal exergy destruction within the physical boundaries of the system as well as the external exergy destruction that occurs in the immediate surroundings due to temperature and concentration gradients that might exist between the physical system and the environment. This requires the exergy analysis of the *extended system*, which is the physical system plus its immediate surroundings that are influenced by the process.

The *extended system analysis* is emphasized in this paper and explicit exergy efficiency relations are developed for the extended systems as well as the physical systems. Extended system analysis is more meaningful because it provides a more realistic picture of the entire process’s thermodynamic performance rather than just what occurs within the device. It is also much simpler since *exergy losses* associated with *heat loss* and with *matter purged* into the environment, if any, are zero. All *external irreversibilities* are properly accounted for as part of the exergy destruction that occurs during the process.

In general, the *exergy efficiency* of the extended system will be lower than that of the physical system since the exergy destroyed term in the former includes exergy destruction within the immediate surroundings as well as within the physical system. The *fraction* of external exergy destruction can be determined by calculating the exergy associated with heat loss to the environment and purged mass and dividing it by the exergy destruction determined for the extended system. With extended system analysis, determining the exergy efficiencies of devices with heat loss and purged mass is as straightforward as determining them for adiabatic devices. In the absence of purged mass, the exergy efficiency relations for adiabatic devices and extended systems become identical.

When determining exergy efficiency, it is important to correctly identify what constitutes expended as well as recovered exergy. For steady-flow devices, the exergy of the fluid stream may increase or decrease during the process depending on whether the fluid stream supplies exergy or recovers exergy as it flows through the device. Making this distinction results in two general exergy efficiency relations for exergy efficiency for a general steady-flow process as follows:

$$\mathrm{For}\;\Delta {\dot{X}}_{\mathrm{mass}}\;<\;0:\;\eta_{\mathrm{ex}}=\frac{{\dot{X}}_{\mathrm{recovered}}}{{\dot{X}}_{\mathrm{expended}}}=\frac{{\dot{X}}_{W\_\mathrm{out}}+{\dot{X}}_{Q\_out}}{{\dot{X}}_{W\_\mathrm{in}}+{\dot{X}}_{Q\_in}+{\dot{(X}}_{1}-{\dot{X}}_{2})}$$

$$\mathrm{For}\;\Delta {\dot{X}}_{\mathrm{mass}}\;>\;0:\;\eta_{\mathrm{ex}}=\frac{{\dot{X}}_{\mathrm{recovered}}}{{\dot{X}}_{\mathrm{expended}}}=\frac{{\dot{X}}_{W\_\mathrm{out}}+{\dot{X}}_{Q\_out}+{\dot{(X}}_{2}-{\dot{X}}_{1})}{{\dot{X}}_{W\_\mathrm{in}}+{\dot{X}}_{Q\_in}}$$

These relations can also be used for the *extended* systems by setting $\dot{X}$*_Q_*__out_ = 0. For *adiabatic* work-producing devices such as turbines and work-consuming devices such as compressors, the relations above simplify as follows:

$$\eta_{\mathrm{ex},\text{work-producing},\mathrm{adiabatic}}=\frac{{\dot{X}}_{W\_\mathrm{out}}}{{\dot{X}}_{1}-{\dot{X}}_{2}}=\frac{{\dot{W}}_{\mathrm{act},\mathrm{out}}}{{\dot{W}}_{\mathrm{rev},\mathrm{out}}}$$

$$\eta_{\mathrm{ex},\text{work-consuming},\mathrm{adiabatic}}=\frac{{\dot{X}}_{2}-{\dot{X}}_{1}}{{\dot{X}}_{W\_\mathrm{in}}}=\frac{{\dot{W}}_{\mathrm{rev},\mathrm{in}}}{{\dot{W}}_{\mathrm{act},\mathrm{in}}}$$

For heat engines, refrigerators, and heat pumps, it is shown that the exergy efficiency relations that involve exergy input–output, exergy expended–recovered, exergy destroyed, reversible and actual work, and actual and reversible work are all equivalent to each other and give the same result for exergy efficiency.

Heat engines:

$$\eta_{\mathrm{ex}}=\frac{{\dot{X}}_{\mathrm{recovered}}}{{\dot{X}}_{\mathrm{expended}}}=\frac{{\dot{X}}_{\mathrm{output}}}{{\dot{X}}_{\mathrm{input}}}=1-\frac{{\dot{X}}_{\mathrm{destroyed}}}{{\dot{X}}_{\mathrm{expended}}}=\frac{{\dot{W}}_{\mathrm{act},\mathrm{out}}}{{\dot{W}}_{\mathrm{rev},\mathrm{out}}}=\frac{\eta_{\mathrm{th},\mathrm{act}}}{\eta_{\mathrm{th},\mathrm{rev}}}$$

Refrigerators:

$$\eta_{\mathrm{ex}}=\frac{{\dot{X}}_{\mathrm{recovered}}}{{\dot{X}}_{\mathrm{expended}}}=\frac{{\dot{X}}_{\mathrm{output}}}{{\dot{X}}_{\mathrm{input}}}=1-\frac{{\dot{X}}_{\mathrm{destroyed}}}{{\dot{X}}_{\mathrm{expended}}}=\frac{{\dot{W}}_{\mathrm{rev},\mathrm{in}}}{{\dot{W}}_{\mathrm{act},\mathrm{in}}}=\frac{{COP}_{R,\mathrm{act}}}{{COP}_{R,\mathrm{rev}}}$$

Heat pumps:

$$\eta_{\mathrm{ex}}=\frac{{\dot{X}}_{\mathrm{recovered}}}{{\dot{X}}_{\mathrm{expended}}}=\frac{{\dot{X}}_{\mathrm{output}}}{{\dot{X}}_{\mathrm{input}}}=1-\frac{{\dot{X}}_{\mathrm{destroyed}}}{{\dot{X}}_{\mathrm{expended}}}=\frac{{\dot{W}}_{\mathrm{rev},\mathrm{in}}}{{\dot{W}}_{\mathrm{act},\mathrm{in}}}=\frac{{COP}_{\mathrm{HP},\mathrm{act}}}{{COP}_{\mathrm{HP},\mathrm{rev}}}$$

Depending on what is known, the most suitable relation can be used to determine the exergy efficiency of a cyclic device.

The comprehensive explicit relations developed for exergy efficiency of steady-flow devices and the insights provided are expected to contribute to the second-law analysis of energy systems and bring consistency and uniformity to the analysis.

## Data Availability

No new data were created or analyzed in this study. Data sharing is not applicable to this article.

## Nomenclature

COP	coefficient of performance
*e*	specific energy (kJ/kg)
*g*	gravitational acceleration (m^2^/s)
*g*	Gibbs function (kJ/kg)
*h*	enthalpy (kJ/kg)
ke	kinetic energy (kJ/kg)
*m*	mass (kg)
*N*	mole number (kmol)
*P*	pressure (kPa)
pe	potential energy (kJ/kg)
*Q*	heat transfer (kJ)
*R*	gas constant (kJ/kg·K)
*s*	entropy (kJ/kg·K)
*T*	temperature (K)
*u*	internal energy (kJ/kg)
*v*	specific volume (m^3^/kg)
*V*	velocity (m/s)
*W*	work (kJ)
*X*	exergy (kJ)
*y*	mole fraction
*z*	elevation (m)
Greek letters	
*η*	efficiency
*μ*	chemical potential (kJ/kg)
*ρ*	density (kg/m^3^)
Subscripts	
0	environmental state
act	actual
*b*	boundary
ch	chemical
env	environment
ex	exergy
gen	generation
*H*	high temperature
*i*	component *i*
in	input
*m*	mixture
*L*	low temperature
*p*	product
out	output
*r*	reactant
*R*	reservoir
rev	reversible
surr	surroundings
sys	system
Superscipts	
˙	time rate
¯	per unit mole
^0^	standard reference state

## References

[1] Çengel Y.A., Boles M.A., Kanoğlu M. (2024). Thermodynamics: An Engineering Approach.

[2] Moran M.J., Shapiro H.N., Boettner D.D., Bailey M.B. (2024). Fundamentals of Engineering Thermodynamics.

[3] Borgnakke C., Sonntag R.E. (2017). Fundamentals of Thermodynamics.

[4] Bejan A., Tsatsaronis G., Moran M.J. (1995). Thermal Design and Optimization.

[5] Bejan A. (2016). Advanced Engineering Thermodynamics.

[6] Kotas T.J. (1985). The Exergy Method of Thermal Plant Analysis.

[7] Szargut J., Morris D.R., Steward F.R. (1988). Exergy Analysis of Thermal, Chemical and Metallurgical Processes.

[8] Szargut J. (2005). Exergy Method: Technical and Ecological Applications.

[9] Wark K. (1988). Thermodynamics.

[10] Moran M.J. (1989). Availability Analysis: A Guide to Efficient Energy Use.

[11] Rant Z. (1956). Exergie, ein neues Wort für “technische Arbeitsfähigkeit” (Exergy, a new word for ‘technical work capacity’). Forsch. Ing. Wes..

[12] Sciubba E., Wall G. (2007). A brief commented history of exergy from the beginnings to 2004. Int. J. Thermodyn..

[13] Cornelissen R.L. (1997). Thermodynamics and Sustainable Development: The Use of Exergy Analysis and the Reduction of Irreversibility. Ph.D. Thesis.

[14] Brodyansky V.M., Sorin M.V., Le Goff P. (1994). The Efficiency of Industrial Processes: Exergy Analysis and Optimization.

[15] Lior N., Zhang N. (2007). Energy, exergy, and Second Law performance criteria. Energy.

[16] Marmolejo-Correa D., Gundersen T. (2012). A comparison of exergy efficiency definitions with focus on low temperature processes. Energy.

[17] Nguyen T.V., Voldsund M., Elmegaard B., Ertesvåg S.I. (2014). On the definition of exergy efficiencies for petroleum systems: Application to offshore oil and gas processing. Energy.

[18] Paul A., Panua R., Debroy D. (2017). An experimental study of combustion, performance, exergy and emission characteristics of a CI engine fueled by Diesel-ethanol-biodiesel blends. Energy.

[19] Chen Q., Mengqi Y., Gang Y., Jianlin Y. (2022). Thermodynamic analyses of a modified ejector enhanced dual temperature refrigeration cycle for domestic refrigerator/freezer application. Energy.

[20] Gurbuz E.Y., Kecebas A., Sozen A. (2022). Exergy and thermoeconomic analyses of the diffusion absorption refrigeration system with various nanoparticles and their different ratios as work fluid. Energy.

[21] Molina-Salas A., Quiros C., Gigant P., Huertas-Fernández F., Clavero M., Monino A. (2023). Exergy assessment and sustainability of a simple off-shore oscillating water column device. Energy.

[22] Kirmizi M., Aygun H., Turan O. (2024). Stage-based exergy analysis for a modern turboprop engine under various loading. Energy.

[23] Kanoglu M. (2002). Exergy analysis of a dual-level binary geothermal power plant. Geothermics.

[24] Kanoglu M. (2002). Exergy analysis of multistage cascade refrigeration cycle used for natural gas liquefaction. Int. J. Energy Res..

[25] Onigbajumo A., Taghipour A., Will G., Van T.C., Couperthwaite S., Steinberg T., Rainey T. (2022). Effects of process-thermal configuration on energy, exergy, and thermo-economic performance of solar driven supercritical water gasification. Energy Convers. Manag..

[26] Calise F., d’Accadia M.D., Macaluso A., Piacentino A., Vanoli L. (2016). Exergetic and exergoeconomic analysis of a novel hybrid solar-geothermal polygeneration system producing energy and water. Energy Convers. Manag..

[27] Ren C., Wang J., Chen H., Liu X., An M. (2021). Thermodynamic analysis and comparative investigation of a new combined heating and power system driving by medium-and-high temperature geothermal water. Energy Convers. Manag..

[28] Vandani A.M.K., Joda F., Boozarjomehry R.B. (2016). Exergic, economic and environmental impacts of natural gas and diesel in operation of combined cycle power plants. Energy Convers. Manag..

[29] Naserian M.M., Farahat S., Sarhaddi F. (2017). New exergy analysis of a regenerative closed Brayton cycle. Energy Convers. Manag..

[30] Urbanucci L., Testi D. (2019). Integration of reversible absorption heat pumps in cogeneration systems: Exergy and economic assessment. Energy Convers. Manag..

[31] Hu Z., de León F., Wang R., Li Y. (2023). Effects of Installing Different Types of Cooling Fins on the Cold Side of a Thermoelectric Power Generation Device on the Thermal Efficiency and Exergy Efficiency of Power Cable Surface Waste Heat Recovery. Micromachines.

[32] Brötz J., Schanzle C., Pelz P.F. (2023). Exergy-based efficiency assessment of fans vs. isentropic efficiency. Int. J. Turbomach. Propuls. Power.

[33] Zulkefal M., Ayub A., Sethi H. (2024). Exergy analysis of methanol production plant from hydrogenation of carbon dioxide. Mater. Proc..

[34] Hecht K., Reboso A.O., van der Vegt M., Appelman J., Zari M.P. (2024). Ecologically regenerative building systems through exergy efficiency: Designing for structural order and ecosystem services. Land.

[35] Zhao Z., Wang Z., Wang H., Zhu H., Xiong W. (2023). Conventional and Advanced Exergy Analyses of Industrial Pneumatic Systems. Energies.

[36] Ghorbani B., Mehrpooya M., Hamedi M.H., Amidpour M. (2017). Exergoeconomic analysis of integrated natural gas liquids (NGL) and liquefied natural gas (LNG) processes. Appl. Therm. Eng..

[37] Taner L. (2015). Optimisation processes of energy efficiency for a drying plant: A case of study for Turkey. Appl. Therm. Eng..

[38] Aijundi I.H. (2009). Energy and exergy analysis of a steam power plant in Jordan. Appl. Therm. Eng..

[39] Bi Y.H., Wang X.H., Liu Y., Zhang H., Chen L.G. (2009). Comprehensive exergy analysis of a ground-source heat pump system for both building heating and cooling modes. Appl. Energy.

[40] Xu K., Qi Y., Sun C., Ai D., Wang J., He W., Yang F., Ren H. (2024). Exergy Flow as a Unifying Physical Quantity in Applying Dissipative Lagrangian Fluid Mechanics to Integrated Energy Systems. Entropy.

[41] Wall G. (1977). Exergy-A Useful Concept Within Resource Accounting.

[42] Righetto F.G., Mady C.E.K. (2023). Exergy Analysis of a Sugarcane Crop: A Planting-to-Harvest Approach. Sustainability.

[43] Abusoglu A., Kanoğlu M. (2008). First and second law analysis of diesel engine powered cogeneration systems. Energy Convers. Manag..

[44] Kanoğlu M., Dincer I., Rosen M.A. (2007). Understanding energy and exergy efficiencies for improved energy management in power plants. Energy Policy.

[45] Rosen M.A., Dincer I., Kanoğlu M. (2008). Role of exergy in increasing efficiency and sustainability and reducing environmental impact. Energy Policy.

[46] Kanoğlu M., Çarpinlioglu M.O., Yıldırım M. (2004). Energy and Exergy Analyses of an Experimental Open-Cycle Desiccant Cooling System. Appl. Therm. Eng..

[47] Wang D., Zhang X., Zhu Y., Jiang Z. (2024). Optimization of Exergy Efficiency in a Walking Beam Reheating Furnace Based on Numerical Simulation and Entropy Generation Analysis. Processes.

[48] Wall G. (2003). Exergy tools. Proc. Inst. Mech. Eng. Part A J. Power Energy.

[49] Magnanelli E., Berglihn O.T., Kjelstrup S. (2018). Exergy-based performance indicators for industrial practice. Int. J. Energy Res..

[50] Lozano M.A., Valero A. (1993). Theory of exergetic cost. Energy.

[51] Lazzaretto A., Tsatsaronis G. (2006). SPECO: A systematic and general methodology for calculating efficiencies and costs in thermal systems. Energy.

[52] Szargut J. (1989). Chemical exergies of the elements. Appl. Energy.

[53] Tsatsaronis G. (2007). Definitions and nomenclature in exergy analysis and exergoeconomics. Energy.

[54] Bejan A. (2002). Fundamentals of exergy analysis, entropy-generation minimization and the generation of flow architecture. Int. J. Energy Res..

[55] Çengel Y.A., Kanoğlu M. (2025). Exergy Efficiency of Closed and Unsteady-Flow Systems. Entropy.

[56] Dincer I., Çengel Y.A. (2001). Energy, entropy and exergy concepts and their roles in thermal engineering. Entropy.

[57] Hepbasli A. (2008). A key review on exergetic analysis and assessment of renewable energy resources for a sustainable future. Renew. Sustain. Energy Rev..

[58] (2025). Engineering Equation Solver (EES) Software.

